# Persistence and conspecific observations improve problem-solving abilities of coyotes

**DOI:** 10.1371/journal.pone.0218778

**Published:** 2019-07-10

**Authors:** Julie K. Young, Laura Touzot, Stacey P. Brummer

**Affiliations:** 1 USDA-National Wildlife Research Center-Predator Research Facility, Millville, Utah, United States of America; 2 Department of Wildland Resources, Utah State University, Logan, Utah, United States of America; 3 Department of Biology, University of Grenoble Alpes, Grenoble, France; Memorial University of Newfoundland, CANADA

## Abstract

Social learning has important ecological and evolutionary consequences but the role of certain factors, such as social rank, neophobia (i.e., avoidance of novel stimuli), persistence, and task-reward association, remain less understood. We examined the role of these factors in social learning by captive coyotes (*Canis latrans*) via three studies. Study 1 involved individual animals and eliminated object neophobia by familiarizing the subjects to the testing apparatus prior to testing. Studies 2 and 3 used mated pairs to assess social rank, and included object neophobia, but differed in that study 3 decoupled the food reward from the testing apparatus (i.e., altered task-reward association). For all three studies, we compared performance between coyotes that received a demonstration from a conspecific to control animals with no demonstration prior to testing. Coyotes displayed social learning during study 1; coyotes with a demonstrator were faster and more successful at solving the puzzle box but did not necessarily use the same modality as that observed to be successful. In study 2, there was no difference in success between treatment groups but this is likely because only one coyote within each pair was successful so successful coyote results were masked by their unsuccessful mate. In study 3, there was no difference in success between treatment groups; only two coyotes, both dominant, hand-reared males with demonstrators were able to perform the task. However, coyotes with a demonstrator were less neophobic, measured as latency to approach the object, and more persistent, measured as time spent working on the apparatus. Social rank was the best predictor of neophobia and persistence and was also retained in the best model for time to eat inside the apparatus, a post-trial measurement of object neophobia. These results suggest coyotes are capable of social learning for novel tasks but social rank, neophobia, and persistence influence their social-learning capabilities. This study contributes to understanding the mechanisms underlying how animals gain information about their environment.

## Introduction

An animal’s ability to learn a new behavior is important to its success in solving ecological problems inherent in a changing environment. Novel behavior, generated through innovation or acquired through use of social information, allows animals to swiftly adapt to shifting niches and variable resource availability [1–2]. Social learning, the transfer of information among individuals through observation and/or interaction [3–4], is of particular interest as it allows innovative solutions to spread throughout a group [1,5]. Social learning might be mediated by a variety of mechanisms such as stimulus enhancement, social facilitation, emulation, and imitation [2,3,6]. Using any of these mechanisms can be advantageous because social learning may reduce the time and effort needed for individual learning [7–9] when the learner can benefit from the experience and mistakes of others [6].

Despite the potential benefits of social learning, individuals may or may not utilize information displayed by conspecifics [10]. Inherent individual characteristics such as sex and age influence the use of socially available information [11]. Several studies show that certain characteristics, such as social rank and neophobia (i.e., avoidance of novel objects), also influence whether an individual chooses to copy a conspecific, independently of whether it is advantageous to do so [12–13]. In fact, the impact of social rank on social learning is multifaceted. Dominant individuals may physically interfere with subordinates attempting a socially learned task [14] or monopolize the necessary resources [15]. Further, social learning may be influenced by the identities of both the demonstrator and the observer. In a variety of species, subordinate individuals will pay more attention to dominant demonstrators [16–18]. Alternatively, this may simply reflect a lack of need-based motivation [19], differences among personalities [20], or a combination of personality with other traits [21]. Conversely, evidence suggests that some dominant individuals use socially learned information more effectively than subordinates, although the mechanism is unclear. This difference in information use may be related to differences in attentiveness between social ranks [15].

In addition to the effects of social rank, neophobia may impede both asocial and social learning, and exploratory behavior [22–24]; however, some studies do not show a relationship between neophobia and exploration [25–27]. In many cases, less neophobic individuals are more likely to interact with novel objects, food, or environments and therefore likely to solve problems via asocial learning [22,24,28]. However, individuals that are more neophobic may use social learning more frequently because the willingness of bolder individuals to explore novel resources provides opportunity for more neophobic animals to learn from inadvertent demonstrations [29]. Neophobia may also be context specific and, therefore, its influence on social learning may vary. For example, demonstrator cues are used to consume novel food but not used to approach a novel object outside of breeding season in jackdaws (*Corvus monedula*) [30].

An individual’s performance of a socially learned task is also influenced by persistence. Persistence is commonly investigated in relation to innovative behaviors [31] and personality [32], and some evidence suggests that it also has a role in successful social learning [33,34]. Hoppit et al. [34] describe ‘observational perseverance’ as an individual’s increased level of persistence of working on a novel task following the successful demonstration of that task. Several studies have investigated persistence by comparing performance between a solvable and unsolvable task [35,36]. The difference in persistence between successful and unsuccessful observers in solvable and unsolvable tasks, especially when considered in the context of social rank or neophobia, remains an underrepresented area of research.

Finally, task performance can also be influenced by saliency of the reward. In many task performance studies, a reward is directly associated with the task. However, the reward can also be decoupled from the task, creating a delay in time and/or space. These non-associative tests are typically used to examine social learning through imitation [37]. This type of learning response does not always result in exact imitation of the way in which the task is solved, but simply any response facilitation [38], where the subject detects and encodes a perceived action and produces an action that has a clear similarity. However, most tests to date have used animals imitating human actions and only recently has it been shown that animals may learn novel tasks using this form of decoupled food reward [39,40]. Tests to measure this type of learning further informs our understanding of animal cognition [41].

Here, we used a captive population of coyotes (*Canis latrans*) to study social learning in a controlled and directly observable setting. Coyotes, exhibit a well-defined social hierarchy in which an individual’s social rank determines its priority of access to resources [42–44]. Coyotes are also generalist foragers [44] that occasionally cache food and dig den sites. They are highly adaptable to a number of environments. Coyote populations continue to expand farther into urban and suburban areas [45], potentially risky and dynamic environments with variable resources, despite the characterization of being notoriously neophobic animals [46,47]. In canids, enhancement of performance due to observations of conspecific behavior may be key in their adaptation to unpredictable environments [48,49]. Thus, coyotes provide an ideal biological model to explore social learning, the influence of mitigating factors, such as social rank, and task persistence in a species that shows behavioral flexibility when faced with variable environments [50].

In the present study, we aimed to examine social learning and task persistence of coyotes. We first set out to determine if coyotes could learn to perform a novel task through social learning, further described as study 1. We then investigated the influence of inherent individual characteristics (i.e., sex and social rank) in determining problem-solving success for coyote dyads after watching a demonstrator perform a task with a salient food reward, described as study 2. This was repeated but with a decoupled food reward, described as study 3. We investigated the differences in success rate, neophobia, latency to solve the task, and persistence in task performance between observers and controls in all three studies.

In accordance with previous studies, we hypothesized that coyotes are capable of social learning and would therefore be more successful and faster at performing a novel task after watching a conspecific demonstrator compared to coyotes that did not observe a demonstrator coyote perform the task. We also expected that persistence, the time coyotes spent working on the problem (see Table 1 for a complete description of the behaviors described and studied throughout the manuscript), would be greater in coyotes that observed a demonstrator and would correlate with successful task performance across all three tasks. We predicted that success would increase across trials (i.e., via individual learning) and that object neophobia, measured as the latency to approach the novel task (Table 1), would be lower in coyotes that observed a demonstrator and in those given an opportunity to interact with the device prior to testing (i.e., study 1). We expected social hierarchy to impact individual behavior in studies 2 and 3 such that dominant individuals within each pair would be more persistent and less neophobic. Thus, we expected dominant coyotes to have higher problem-solving success rates.

**Table 1:** Designation, description, and measurement of the behaviors observed during three studies on social learning in captive coyotes.

Behaviour	Description of the behavior	Measurement of the behavior
**Neophobia**	Avoidance or fear of a novel stimuli.	*Latency to approach*: willingness of the individual to engage with the novel object, by eating a food bait in (study 1) or entering a 1-m radius around the novel object (studies 2 and 3).
**Persistence**	The novel object holds the attention of the individual and the subject interacts with it. Indirection interactions include looking at the object, walking around without any contact, and sniffing/digging/scratching next to the hoop. Direct interactions include investigating the ground in a close perimeter (i.e. less than a body-length) by smelling/searching/digging, investigating the novel object by pushing/pulling/biting it, and going on/into the novel object (i.e. stepping into the hoop).	*Persistence/Working time*: the amount of time spent engaged with the puzzle box (i.e. muzzle, paw or body within 17cm; study 1) or the time during which the coyote interacted directly or indirectly with the hoop by performing one of the relevant behavior defined (studies 2 and 3). Neophobia was not considered persistence.
**Attentiveness**	The observer watching its assigned demonstrator.	*Attentiveness*: time the observer spent oriented toward its assigned demonstrator.
**Other**	Any other behavior that is not linked to the apparatus (e.g. interacting with the neighbor, walking randomly in the pen, scratching).	No direct measurement taken; sum of remaining time.

## Materials and methods

### Study site

**Fig 1:**
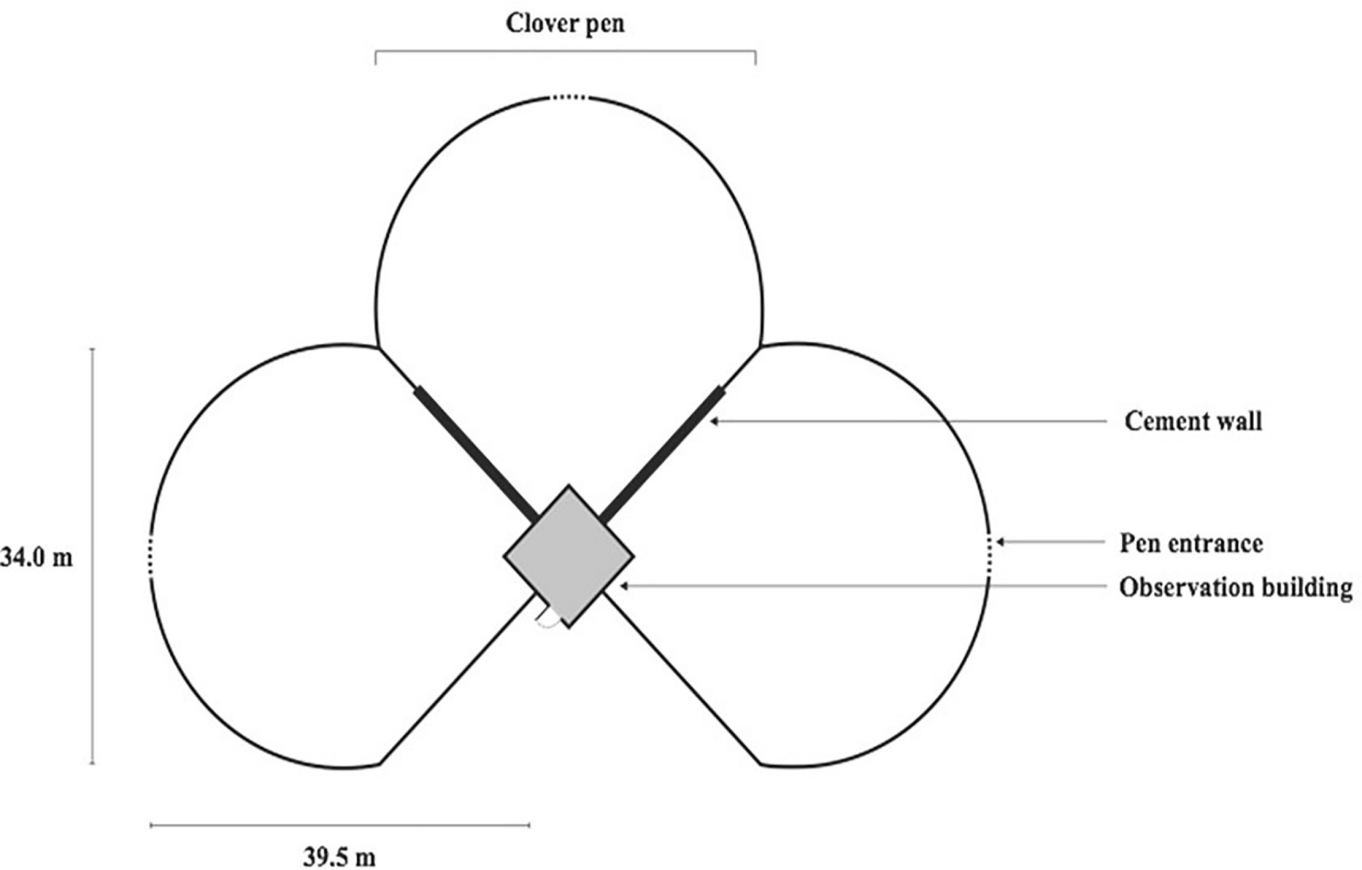
Schematic depicting three of the 1000 m^2^ enclosures housing single or male-female pairs of coyotes within a single housing block at the USDA-National Wildlife Research Center’s Predator Research Facility (Millville, UT, USA). The enclosures are tear-drop shaped, adjoined by a common observation building at the narrow ends. Subjects could see immediate neighbors through the chain-link fence at the wider end of the enclosure but could not see neighbors towards the narrow end because of cement walls abutting the observation building. A plywood fence was erected that extended from the cement wall when needed to prevent neighbors from seeing one another at the wide ends.

Experiments were conducted at the USDA National Wildlife Research Center’s (NWRC) Predator Research Facility in Millville, Utah. The facility maintains about 100 adult coyotes in captivity housed in large outdoor pens (0.1–1.0 ha) as male-female pairs. Most of the coyotes are born and parent-reared on site but some are brought to the facility as wild-born pups to increase genetic diversity within the colony. Wild-born pups are hand reared until 10 weeks of age and then raised similarly to the captive-born coyotes. At approximately 10 months of age, coyotes are placed into male-female pairs that are maintained throughout their lifespan to promote animal welfare and retain behavior observed in wild coyotes (i.e., territorial defense, aggression, pair-bond formation, scent marking) [51]. The coyotes are fed 650g of a commercial mink food (Fur Breeders Agricultural Cooperative Logan, Utah) at least six days each week by a caretaker entering the enclosure and scattering the food. For this study, the investigator running each task fed the coyotes in the weeks leading up to the experiment, so that the coyotes became accustomed to the investigator’s presence. The investigator also ensured that all individuals present in the enclosure ate the food immediately during feeding time (i.e., no latency to approach or eat food). All experiments were conducted before coyotes were fed for the day to ensure similar food motivation [52]. At least 10 days before testing began, all coyotes were housed in 1000 m^2^ outdoor pens used for testing during all trials (Fig 1). This allowed coyotes to investigate the test enclosure in the absence of the testing apparatus and considerably minimized the amount of time spent investigating the enclosure during the first experimental trial [28]. Study 1 took place in March and April of 2011 while studies 2 and 3 were conducted between February and July of 2016.

### Study 1: direct food-reward task for individuals

#### Experimental apparatus

**Fig 2:**
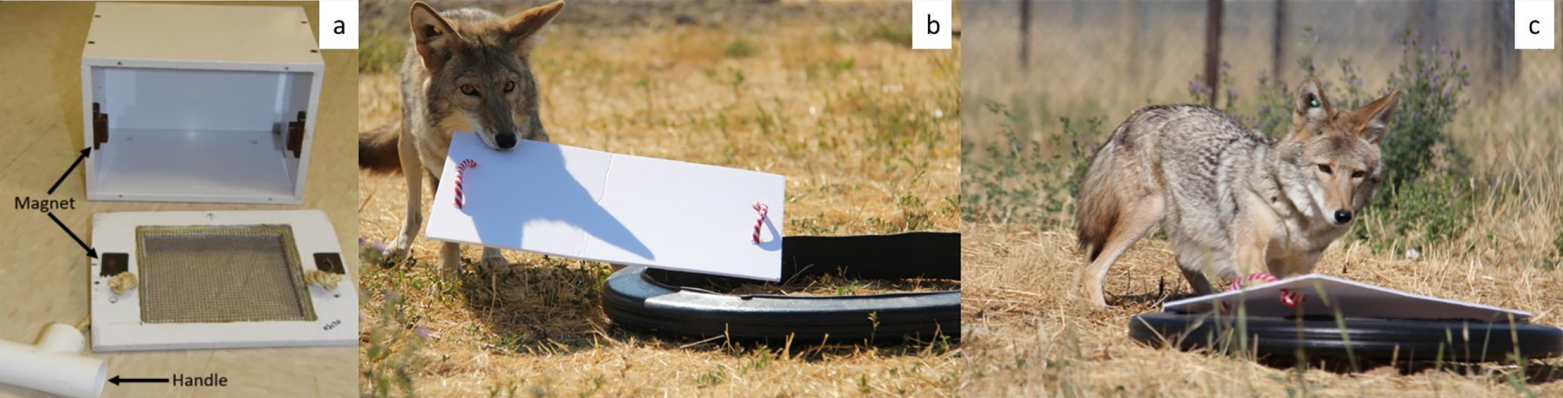
The puzzle box used for task of study 1 (a) and captive coyotes at the USDA-Predator Research Facility performing the task of study 2 (b and c). Observer coyotes opened the puzzle box used in study 1 by pulling on the handles and uncovered the hoop covered with foam board used in study 2 with their muzzle (b) or paws (c).

We used a puzzle-box test to assess whether coyotes could display social learning. The puzzle box was made with white PVC plastic (L: 23 cm x H: 17 cm x W: 16 cm) and had a removable door attached via magnets (Fig 2A); the design allowed the individual to smell and see the food reward inside through a screened door. Two handles were attached on the door, one on each end, to allow coyotes to open the door using either a paw or muzzle. The puzzle box was anchored to the enclosure floor prior to and during the experiment and each tested coyote had its own puzzle box throughout the whole experiment in order to prevent local enhancement due to odor cues deposited by another individual. A coyote was considered successful at solving the task if it ate the food reward inside the puzzle box after removing the door.

#### Subjects

We first identified which adult coyotes (1–7 years old) would interact with a novel enrichment object (i.e., pine tree branch) within an hour to gain access to an extra portion of their food. Using a random block design to balance for sex, we then randomly assigned six of these coyotes to serve as observers and six as controls (Table 2, see S1 Table for additional details). Four additional coyotes that demonstrated both a willingness to interact with a novel object and a high tolerance for human presence (i.e., would manipulate enrichment objects with a person standing in the enclosure) were chosen as demonstrators (Table 2). None of these coyotes were exposed to any manipulative problem-solving tasks prior to this experiment.

**Table 2:** Sample sizes of captive coyotes at the USDA-National Wildlife Research Center’s Predator Research Facility in Millville, Utah, USA, used in three different studies on social learning.

	Study 1	Study 2	Study 3
Number of demonstrator coyotes	4	2	2
Number of control coyotes	6	8 (4 pairs)	8 (4 pairs)
Number of observer coyotes	6	16 (8 pairs)	20 (10 pairs)
Number of successful control coyotes	4	0	0
Number of successful observer coyotes	5	4	2

#### Experimental procedure

The experimental period was made up of five consecutive demonstration days followed immediately by five consecutive trial days. Two (one male and one female) coyotes, assigned as demonstrators for the observer coyotes, were trained to open the puzzle box using successive approximation. Specifically, they were first fed from a puzzle box with the door open to allow access to the food and some food was placed on a door handle to elicit a handle-biting response. Over time, the puzzle door was gradually moved to a closed position and the coyotes learned to open the puzzle box. Coyotes were trained in about three weeks and were considered as demonstrators after consistently performing the task during feeding. Additionally, in an effort to prevent object neophobia from inhibiting task performance (Table 1), all subjects were trained to eat from an open puzzle box with no door prior to the experimental period by successively feeding them closer to the box.

Two other coyotes (one male and one female) were used as demonstrators for the control coyotes to control for differences due to social facilitation. The only requirement for control demonstrators was to eat food from the open puzzle box within 5 minutes with a human investigator nearby; they were not trained to open the puzzle box door. All four demonstrator coyotes were always the opposite sex of observer or control animals, to reduce territorial behavior between same sex individuals that may hinder learning [48]. Each observer and control animal was housed either directly next to or across from the assigned demonstrator or control demonstrator, respectively, during the experimental period so that they could watch demonstrations through the chain-link fence along the large part of the tear-drop shaped pen (Fig 1). The puzzle boxes for the observer and control coyotes were situated at the opposite end of the pen, away from demonstrators and neighbors.

For the entirety of the experimental period, the same investigator would enter each demonstrator or control demonstrator’s enclosure to place food in the puzzle box and attach the puzzle door. The investigator adopted a position while manipulating the puzzle box to prevent coyotes from seeing any human-puzzle box interaction. The food consisted of the coyote’s daily ration divided into two food piles. The larger pile, approximately 450 g, was frozen and placed inside the box. A 200 g pile, referred to as the bait from this point onward, was placed 15 cm in front of the puzzle box. The investigator then left the area to allow for the demonstrations to take place without human presence. The demonstrators performed the previously trained task of removing the puzzle-box door to retrieve food and the control demonstrators performed the task of retrieving food from the open box. Once they ate the food placed inside the puzzle box, the investigator would enter each observer and control coyote enclosure to repeat the process of placing a 450 g frozen food pile in the open puzzle box and 200 g of bait in front of the puzzle box.

During the demonstration period, observer and control coyotes were also habituated to the puzzle box door to prevent neophobic responses toward the door itself. The door was placed in the enclosure, approximately 30 cm from the open box on demonstration day one and removed from the enclosure on demonstration day five. The trial period did not begin until the observer or control individual was willing to eat from the open puzzle box within 10 minutes of the food being placed and, in the case of observer coyotes, the individual had observed its assigned demonstrator for five days. One control and one observer, both one-year-old females, failed to eat the food from the box during the entirety of the five-day demonstration period and were not used in trials.

During the trial period, the procedures for daily puzzle box set up and food placement were identical to those in the demonstration period for demonstrators, controls, and observers. The only difference was that the investigator now attached a puzzle-box door onto each control’s and observer’s puzzle box. Each individual trial lasted four hours. A successful individual was defined as one that opened the puzzle box and ate the food placed inside the box in at least one trial. An unsuccessful individual either failed to engage with the puzzle box or failed to open the door and eat the food from the puzzle box during the entirety of the experiment.

#### Data extraction from videotaped trials

Observer and control coyotes were video recorded using digital cameras (Sony Digital 8 and DCR-SR68 Handycams, and Panasonic SDR-H85) at the transparent section of the fence during each demonstration. All puzzle-box trials (i.e., demonstration and testing trials) were also video recorded. A single observer counted the number of times observer and control coyotes observed their respective demonstrator. The same person extracted detailed behavioral data from all trials of each observer and control coyote using Noldus Observer XT software. Box height (17 cm) was used to determine the subject’s proximity to the puzzle box by markings on a transparency sheet overlaying the monitor screen during video review. Data recorded included whether individuals solved the puzzle box, trial day(s) the puzzle box was solved, latency to eat from the puzzle box (named hereafter latency to solve), and persistence (Table 1). For successful observers, we also recorded the modality (muzzle or paw) they used to solve the puzzle box, and the modality used by their assigned demonstrator. Trial start was defined as when the individual finished eating the bait that was used as an indication of the subject’s willingness to engage with the box. At the end of each trial, any unsolved puzzle boxes were opened, the doors were placed flat on the ground, and the food was removed and placed on the ground near the box. The coyote remained in the pen throughout the testing period.

#### Statistical analyses

We first used generalized linear models (GLMM; with success (Y/N) as the binomial response variable) to determine whether treatment group (i.e., control versus observer) and/or trial number (i.e., effect of repeated measures) was/were the best predictor(s) of problem-solving success. All possible models were tested, from the simplest model including no explanatory variables (i.e., constant model, effect of the intercept only) to the model including the main effects of treatment group and trial number, as well as their two-way interactions. Coyote ID was always included in the models as a random effect to account for individual variability, potentially associated with personalities [53]. We then used a stepwise approach and Akaike Information Criterion corrected for small sample size (AICc) for model selection [54]. The model with the lowest AICc was retained as the best fit [54]. When the AICc difference between two competing models was < 2, we retained the model including the lowest number of parameters according to parsimony rules.

We then used GLMMs to examine how latency to solve the puzzle box (i.e. only the trials during which the nine successful individuals solved the problem were included in the models) and working time changed over successive trials for student versus control coyotes. We included trial number and whether the subject was in the student or control group as fixed covariates to determine if coyotes in these two treatment groups differed in their learning to solve the puzzle box task. Coyote ID was always included as a random effect to account for repeated measures and individual variability [53]. Latency to solve the puzzle-box was log-transformed to achieve normality and the gamma distribution was used as the error structure of the models in which persistence was the response variable. Finally, we used a chi-square test to determine whether observer coyotes that were successful at solving the puzzle box displayed the same modality as their respective demonstrator coyote.

For all three studies, distributions of the response variables were tested prior to running the models using the “fitdistrplus” R package [55] and model assumptions (i.e. normality of the residuals, over-dispersion and heteroscedasticity) were verified after performing model selection. All GLMMs were run using the lme4 package, version 1.1–18.1 in R (version 3.5.2, R Development Core Team, 2011). Thus, all results presented assume that model assumptions were respected (i.e. residuals normally distributed, no over-dispersion or heteroscedasticity) or otherwise are noted. Parameter estimates ± standard errors (SE) are provided for all selected models. All analyses were performed with R (version 3.5.2, R Development Core Team, 2011).

#### Results

**Fig 3:**
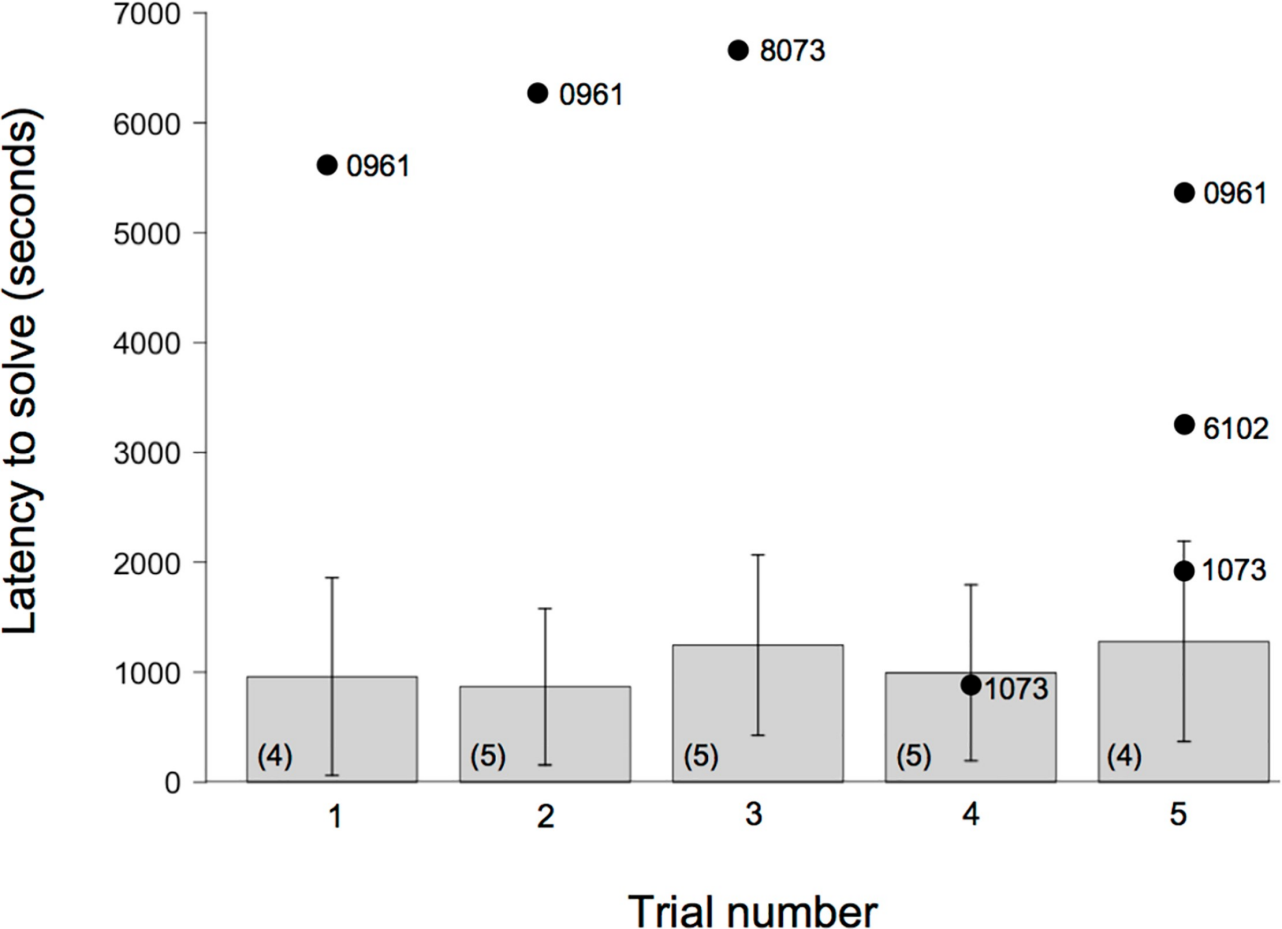
Latency to solve a puzzle box in study 1 for successful observer and control coyotes across all trials. Bar plots show mean ± SE for successful observer coyotes (number of observer coyotes that performed the task successfully on a given trial day is noted within parentheses directly in the bar plot). Sample sizes varied because not all coyotes were successful in every trial. Some control coyotes were also successful. Dots represent latency to solve the task for each successful control coyote in a given trial. ID of the successful control coyote appears directly beside the dot so that repeated successes by the same coyote are easily noted.

All observer coyotes (n = 5) witnessed at least four of the five demonstrations and all control coyotes (n = 5) witnessed at least two, but usually four, of the five control demonstrations before their trials. The probability of success in solving the puzzle box over all trials was best described by the model considering the treatment group of the individuals only (Table 3A; ω = 0.94), observer coyotes being more successful than control individuals (β = 3.33 ± 0.86; Fig 3). While four of five observer coyotes were successful the first day and in subsequent trials, only one of the five control coyotes succeeded in opening the puzzle box and eating the food from the open puzzle box on the first test day (Fig 3).

**Table 3:** Set of models fitted to assess the relationship between treatment group and trial number and (A) problem solving success, (B) latency to solve, and (C) persistence. The error distributions used in the models are specified by model family. Model comparison was based on AICc. Np is the number of parameters, ΔAICc is the difference between the candidate model and the model having the lowest AICc, and ω_i_ is the AICc weight of each model (i.e. measure the likelihood that a given model is the best among the candidate models). Models retained based on the lowest AICc value appears in bold, and models retained based on parsimony rules in italic font.

Model	Model family	Np	AICc	ΔAICc	ω_i_
A) Problem solving success	Binomial				
~ 1 + (1|Coyote ID)		2	58.340	11.166	0.003
** ~ Treatment group + (1|Coyote ID)**	** **	**3**	**47.174**	**0.000**	**0.940**
~ Trial number + (1|Coyote ID)		7	74.552	27.378	1.066
~ Treatment group + Trial number + (1|Coyote ID)		9	52.785	5.611	0.057
~ Treatment group x Trial number + (1|Coyote ID)		12	62.039	14.865	0.000
B) Latency to solve	Gaussian				
~ 1 + (1|Coyote ID)	Log-transformed	2	69.164	2.460	0.226
** ~ Treatment group + (1|Coyote ID)**	** **	**4**	**66.704**	**0.000**	**0.773**
~ Trial number + (1|Coyote ID)		7	83.433	16.279	0.001
~ Treatment group + Trial number + (1|Coyote ID)		9	82.271	15.567	0.000
~ Treatment group x Trial number + (1|Coyote ID)		12	93.912	27.208	0.000
C) Peristence	Gamma				
*~ 1 + (1|Coyote ID)*	* *	*2*	*-71*.*017*	*0*.*481*	*0*.*429*
~ Treatment group + (1|Coyote ID)	** **	4	-71.498	0.000	0.545
~ Trial number + (1|Coyote ID)		7	-64.014	7.884	0.013
~ Treatment group + Trial number + (1|Coyote ID)		9	-63.965	7.533	0.013
~ Treatment group x Tria number + (1|Coyote ID)		12	-55.411	16.087	0.000

Treatment group was also retained by model selection as a predictor of latency to solve the puzzle box (Table 3B; ω = 0.77), unlike trial number. We found that observing a demonstrator significantly decreased the time required to problem solve (β = -1.68 ± 0.82; Fig 3). However, the time coyotes spent engaged with the novel apparatus was not influenced by treatment group or the day the trial took place (Table 3C). Finally, modality used by observer coyotes was independent of modality displayed by their assigned demonstrators to solve the task (X^2^_1_ = 2.23x10^-31^, *P* = 1.000).

These results provide evidence that coyotes are able to learn how to perform a new task through observation of a conspecific. Because it shows that social learning occurs in this species, we were able to continue to studies 2 and 3. However, it is noteworthy that when the door of the puzzle box was successful removed in any trial, most coyotes responded in a way that suggested they were startled (i.e., quickly moved away and/or made quick erratic movements). This consistent response over the course of study 1 led us to modify the apparatus for study 2 and 3 to an object that did not elicit this startle response during successful removals.

### Study 2: direct food-reward task for pairs

#### Experimental procedure

For study 2, we randomly selected 12 male-female pairs of adult coyotes, ranging in age from 1–9 years, from all captive coyotes at the facility to participate. We used a random block design to assign eight pairs as observers (n = 16 observers) and four pairs as controls (n = 8 controls) before the experiment began (Table 2; see S2 Table for detailed information).

We assessed a proxy of social status for each pair via food dominance using a single, 300-g food resource over five winner-loser trials conducted over five consecutive days (S2 Table). This methodology is commonly used for establishing dominance in canids, including coyote [42,46], wolf (*Canis lupus*) [56], and domestic dog (*Canis familiaris*) dyads [57]. Social rank and food dominance have been shown to be related in coyotes [44]. Because coyotes at the facility are housed as male-female pairs, the social rank is only defined within each dyad. Thus, in this study, the coyote within each male-female pair that displaced the other from the food at least four out of five times was defined as dominant and the other as subordinate. Both members of the pair were defined as neutral if neither displaced the other in at least four trials (S2 Table).

The testing apparatus was a black PVC plastic hoop covered with a white polystyrene foam board. Two handles were located on the top side of the foam board to allow coyotes to remove the foam board from the hoop using either a paw or muzzle (Fig 2B and 2C). The experimental apparatus was set-up on the ground around a shallow hole. A frozen food ball (500g) was placed in the hole and slightly buried with loose dirt so that coyotes could smell but not see the food. Thus, a coyote had to remove the board covering the hoop and lightly dig to access a food reward and be considered successful at solving the task.

Three male coyotes selected for their absence of aggressiveness toward the investigator and low level of stress while she/he was inside their enclosure were trained to be demonstrators. Thus, the demonstrators were initially fed next to the hoop, the food was gradually moved inside the hoop, and eventually set in a shallow divot while remaining visible. Over time, the divot was made deeper until the food was placed below ground level. The polystyrene foam board was placed in the enclosure and gradually moved closer to the hoop until it completely covered the hoop. Demonstrator coyotes were used on tests after successfully performing the task three times in a row in < 10 minutes for six consecutive days. Only two met this requirement and were further used as demonstrators.

Pairs of observer coyotes were housed in an enclosure across from their assigned demonstrator. This was a different housing block from the demonstrator but allowed the majority of the transparent chain-link fence section to be visible between pens because the large part of the tear-dropped shaped pens faced one another. The experimental apparatus was placed approximately 2 m from the chain-link fence separating the observer and demonstrator enclosures. Pairs of control coyotes were housed in an enclosure adjacent to the demonstrator’s enclosure, at a similar distance as the observer coyotes, but plywood attached to the outside of the chain-link fence prevented them from seeing the demonstrator assigned to them (Fig 1). All observer and control coyote pairs were food-deprived for 24 hours prior to the start of trails to standardize feeding motivation [52].

One trial was conducted every day for five consecutive days. Observer pairs had the opportunity to watch a demonstrator successfully perform the task to receive a food reward immediately before they were given access to the hoop and board themselves. Trials began when the hoop covered with foam board was set-up inside the demonstrator’s enclosure and the food buried in the divot. The demonstrator performed the task successfully at least three times within each trial, with the investigator covering the hoop with the foam board after each successful performance. In order to reduce the influence of the investigator on behavior of observer coyotes, the manipulation of covering the hoop with the foam board was performed while the observer coyotes were focused on the demonstrator eating the frozen food reward. Additionally, the investigator adopted a squat position, such that the investigator’s back was facing the observers’ enclosure to ensure the observers were always blind towards these actions. Repeated task performance was used because the goal was to ensure the observer coyote saw the task performed at least one time during each day’s trial. Thus, on rare occasions the investigator increased the number of performances of the demonstrator to ensure each observer coyote saw the task being successful performed at least once during each trial. Upon completion of the demonstrations, the investigator immediately removed the experimental apparatus from the demonstrator’s enclosure, placed an identical apparatus inside the enclosure of the observers, and left the enclosure.

The trial ended for each pair of coyotes when one of the subjects performed the task or 30 minutes had passed. The experimental apparatus and food, if the trial was unsuccessful, was immediately removed from the pair’s enclosure at the end of each trial. Control animals experienced the same conditions as observer coyote pairs but had no opportunity to observe conspecifics perform the task before their own trials. Trials for the control coyotes were conducted immediately after the observer’s trials using an identical apparatus and following the same sequence.

For all observer and control pairs, a successful individual was defined as one that moved the board and dug to access food in at least one trial. An unsuccessful individual failed to perform the task in all trials. All coyote pairs that did not perform the task received their daily food rations about 5 hours after a trial ended each day of testing.

A single investigator extracted detailed behavioral data from each trial (i.e. demonstrator, control and observer coyotes) through live observations from an observation vehicle parked approximately 15-m away from the testing enclosure in order to minimize human disturbance effects. Data collected included attentiveness, neophobia, persistence, and latency to solve the task (Table 1). Times for latency measures began when the investigator left the observer’s enclosure.

#### Statistical analyses

We used a four-step approach to evaluate social learning in coyotes in this direct food-reward task for pairs after ensuring model assumptions were met as in study 1. We first used generalized linear mixed models (GLMM; with success (Y/N) as the binomial response variable) to determine whether treatment group (i.e. control versus observer) and/or trial number (i.e., effect of repeated measures) was/were the best predictor(s) of problem-solving success. Coyote ID was included as a random effect to account for individual variability [53]. Trials during which individuals never approached the experimental apparatus, in other words failed to engage with the hoop, were not included in the analyses.

Since all control coyotes failed to problem solve, the next steps of the analyses focused on the observer individuals. First, we used GLMMs (binomial family) to test whether attentiveness, neophobia and/or persistence was/were the best predictor(s) of problem solving success within this treatment group. All variables were tested individually in the models, along with all possible combinations. Coyote ID was always included as a random effect to account for repeated measures. Pair ID was also included as a random effect to account for the non-independence of the behavior displayed within dyads (e.g., an individual preventing its mate to access to the apparatus). All GLMMs were run using the lme4 package (version 1.1–18.1) in R.

Then, we investigated the effect of intrinsic factors, represented by the sex and the social rank of the individuals, on the variable(s) retained as best predictor(s) of problem solving success. Attentiveness, neophobia and persistence, where retained as response variables, had to be log-transformed to achieve normality. Males were significantly more dominant than females (see S1 Fig) so we were unable to properly distinguish between social rank and sex effects. Instead, the interaction between sex and social rank was systematically tested in the models. Both coyote and pair IDs were considered as random effects to account for repeated measures and non-independence of the measures within a pair.

Due to limited sample size, we did not specifically test for an effect of age in the models, but we did account for this potential effect by including age class of the individuals as a random effect (class C refers to coyotes ranging in age from 1 to 2 years of age; class B refers to coyotes 3–6 years of age; class A refers to coyotes ranging in age from 7 to 9 years of age). Finally, we used the same models to examine the effects of habituation (i.e., trial number) on the variable(s) identified as best predictor(s) of problem solving. For all analyses, the model(s) with the lowest AICc was retained as the best fit [54]. When the AICc difference between two competing models was < 2, we retained the model including the lowest number of parameters according to parsimony rules. Parameter estimates ± standard errors (SE) are provided for all selected models.

#### Results

Four observer coyotes (of 8 observer pairs tested) were successful at uncovering the hoop and digging to access the food reward. None of the four pairs of control coyotes solved the direct food reward task. However, problem-solving success was best described by the constant model (i.e., intercept only; Table 4A; ω = 0.607), likely because coyotes were tested as pairs.

**Table 4:** Set of models fitted to assess the relationship between (A) problem solving success, treatment group, and trial number; (B) problem solving success within the observer treatment group and attentiveness, neophobia, and persistence; (C) attentiveness; and (D) persistence within the observer treatment group and sex, social rank, and trial number. The error distributions used in the models are specified by model family. Model comparison was based on AICc. Np is the number of parameters, ΔAICc is the difference between the candidate model and the model having the lowest AICc, and ω_i_ is the AICc weight of each model. Models retained based on the lowest AICc value appears in bold, and models retained based on parsimony rules in italic font.

Model	Model family	Np	AICc	ΔAICc	ωi
A) Problem solving success	Binomial				
** ~ 1 + (1|Coyote ID) + (1|Pair ID)**	** **	**3**	**19.888**	**0.000**	**0.607**
~ Treatment group + (1|Coyote ID) + (1|Pair ID)		4	21.808	1.920	0.232
~ Trial number + (1|Coyote ID) + (1|Pair ID)		7	23.123	3.235	0.120
~ Treatment group + Trial number + (1|Coyote ID) + (1|Pair ID)		9	25.351	5.463	0.039
~ Treatment group x Trial number + (1|Coyote ID) + (1|Pair ID)		12	35.177	15.289	0.000
B) Problem solving success within the observer treatment group	Binomial				
~ 1 + (1|Coyote ID) + (1|Pair ID)		3	33.067	13.833	0.000
~ Attentiveness + (1|Coyote ID) + (1|Pair ID)		3	35.476	16.242	0.000
~ Neophobia + (1|Coyote ID) + (1|Pair ID)		3	31.635	12.401	0.001
~ Persistence + (1|Coyote ID) + (1|Pair ID)		3	20.115	0.881	0.277
~ Attentiveness + Neophobia + (1|Coyote ID) + (1|Pair ID)		4	34.148	14.914	0.001
** ~ Attentiveness + Persistence + (1|Coyote ID) + (1|Pair ID)**	** **	**4**	**19.234**	**0.000**	**0.430**
~ Neophobia + Persistence + (1|Coyote ID) + (1|Pair ID)		4	22.527	3.293	0.083
~ Attentiveness + Neophobia + Persistsence + (1|Coyote ID) + (1|Pair ID)		5	20.691	1.457	0.208
C) Attentiveness within the observer treatment group	Gaussian				
~ 1 + (1|Coyote ID) + (1|Pair ID) + (1|Age class)	Log-transformed	3	225.640	5.309	0.066
** ~ Sex/Social rank + (1|Coyote ID) + (1|Pair ID) + (1|Age class)**	** **	**5**	**220.331**	**0.000**	**0.934**
~ 1 + (1|Coyote ID) + (1|Pair ID) + (1|Age class)		3	225.640	15.486	0.000
** ~ Trial number + (1|Coyote ID) + (1|Pair ID) + (1|Age class)**	** **	**8**	**210.154**	**0.000**	**1.000**
D) Persistence within the observer treatment group	Gaussian				
~ 1 + (1|Coyote ID) + (1|Pair ID) + (1|Age class)	Log-transformed	3	273.345	3.849	0.127
** ~ Sex/Social rank + (1|Coyote ID) + (1|Pair ID) + (1|Age class)**	** **	**5**	**269.496**	**0.000**	**0.873**
~ 1 + (1|Coyote ID) + (1|Pair ID) + (1|Age class)		3	273.345	22.737	0.000
** ~ Trial number + (1|Coyote ID) + (1|Pair ID) + (1|Age class)**	** **	**8**	**250.608**	**0.000**	**1.000**

When focusing on the observer treatment group, both attentiveness and persistence were retained by model selection as predictors of problem solving success (Table 4B; ω = 0.430). Individuals spending more time watching a demonstrator (Table 4C) and working on the novel apparatus (Table 4D) were thus found to be more successful (β = 1.39 ± 0.52 and β = 4.48 ± 1.79 respectively). Indeed, successful observer coyotes spent on average 54.3 ± 4.2% and 77.0 ± 15.6% of the total demonstration and trial times respectively watching their demonstrator and working on the novel apparatus, while unsuccessful observer coyotes only spent 29.2 ± 19.8% and 18.8 ± 8.1% of trial time toward their demonstrator and working on the apparatus. Interestingly, we also observed that attentiveness decreased across trials (Table 4C; e.g., β = -3.81 ± 2.49 and β = -5.53 ± 2.73 for trials 3 and 5 respectively) while persistence increased (Table 4D; e.g., β = 4.17 ± 2.15 and β = 5.13 ± 3.45 for trials 2 and 4 respectively).

Within the observer treatment group, all male coyotes were also the dominant individual within the pair. Thus, we were unable to disentangle between an effect of social rank or sex on predictors of problem solving success. However, we found that sex and/or social rank was/were significant predictors of both attentiveness and persistence (Table 4C and 4D), with males and/or dominant individuals spending more time towards their assigned demonstrator (β = 8.36 ± 2.47) and working on the apparatus (β = 4.04 ± 2.16).

### Study 3: indirect food reward task for pairs

#### Experimental procedure

For Study 3, captive coyotes were presented with the same black PVC plastic hoop used in Study 2, however, no foam board covered the hoop and no food was placed inside. Dirt was still mounded inside the hoop area and coyotes had to display digging behavior inside the hoop area to receive an indirect reward food from a human investigator standing 10-m away from the experimental apparatus. Coyotes received 50g balls of frozen food immediately upon performing the digging behavior. Successful task performance was defined as a coyote digging inside the hoop in the presence of a human.

We attempted to train three male coyotes that exhibited both observant behavior and a high tolerance for human presence in their enclosures to dig within the hoop to obtain the food reward using successive steps. First, the coyotes were fed within the hoop and rewarded when they entered the hoop area with additional food balls. The food was gradually moved inside a hole, and covered with loose soil to elicit the digging behavior. Coyotes were rewarded when entering the hoop area and displaying the digging behavior. Over time, only the digging behavior was rewarded, and the amount of food placed inside the hoop was decreased until no food was placed within the hoop and all food was given as 50g balls by the investigator. We used a clicker device to indicate correct behavior to the demonstrator and 50g balls of frozen food were simultaneously given as a reward to positively reinforce the desired behavior [58]. Two male coyotes were successfully trained over a 1-month period of time and considered as demonstrators when they successfully performed the task three times in a row during each trial for 6 consecutive days within 10 minutes or less.

We used a random block design to assign 14 coyote pairs to observer or control groups (Table 1). Ten pairs of coyotes had the opportunity to watch one of the two demonstrators successfully perform the task to receive a food reward immediately before their trials (n = 20 observers; S3 Table). Four pairs of coyotes served as controls and did not have any opportunity to observe conspecifics perform the task before their trials (n = 8 observers; S2 Table). Because the number of coyotes available at the time of this test was limited by other facility research needs, one female coyote used in study 3 was also used in study 1 (0900; S2 Table). Housing conditions, social status assessments, and experimental procedures were identical to those used in study 2.

While a successful individual was defined as one that entered the hoop area and dug to receive a food reward in at least one trial, an unsuccessful individual was defined as one who failed to perform the task in all trials. All coyotes who did not perform the task were fed about five hours after the trial. Unsuccessful observer and control coyotes were subject to an additional trial at the end of the five testing days in which 650 g of fresh food was placed within the hoop to determine if object neophobia may have caused failure.

A single investigator extracted detailed behavioral data from each trial through direct observations from within the enclosure. Data recorded were identical to study 2, with the addition of latency to eat food from the hoop in the post-trial test of unsuccessful pairs of coyotes.

#### Statistical analyses

Considering the high rate of unsuccessful individuals (92.9%), we focused statistical analysis on the effects of social learning opportunities and individual characteristics on persistence and neophobia. Thus, after we ensured that model assumptions were met as in study 1, we used GLMMs (lme4 package in R) to assess the effects of treatment group and trial number on these two variables. Coyote and pair IDs were included as random effects to account for repeated measures (i.e., trials) and non-independence of the measures between members of a pair. Trials during which individuals failed to approach the experimental apparatus were not included in the analyses. Latency to approach and time spent working on the problem were both log-transformed to achieve normality. We then focused the analyses on the observer treatment group to determine whether individual characteristics captured by sex and social rank influenced both neophobia and persistence. Age class was included as a random effect to account for a potential age effect.

Finally, we used the same approach to inquire how latency to eat inside the hoop in the post-trial test was influenced by treatment group, sex, social rank, and the interaction between these two intrinsic factors of the unsuccessful coyotes. The gamma distribution was used as the error structure in the models with latency to eat in the post-trial test as the response variable, and age class of the individuals was added as a random effect to account for this inherent individual characteristic along with pair ID. The model with the lowest AICc was retained as the best fit [54]. When the AICc difference between two competing models was < 2, we retained the model including the lowest number of parameters according to parsimony rules. Parameter estimates ± standard errors (SE) are provided for all selected models.

#### Results

Of the 14 pairs tested with the hoop, two observer coyotes from two different pairs entered the hoop area and dug to receive a food reward in at least one trial (7.1%). They also solved the task multiple times. Both were two years old, hand-reared as pups, and dominant within their pair. Despite their success, neither treatment group nor trial number were predictors of problem-solving success (Table 5A; constant model retained as best model). The 12 pairs where neither individual solved the task also failed to engage directly with the hoop.

**Table 5:** Set of models fitted to assess the relationship between (A) problem solving success, (B) neophobia and (D) persistence and treatment group and trial number, (C) neophobia, and (E) persistence within the observer treatment group and individual characteristics (sex and social rank), and (F) time to eat inside the hoop for unsuccessful coyotes in the post-trial tests and treatment group, sex, and social rank. The error distributions used in the models are specified by model family. Model comparison was based on AICc. Np is the number of parameters, ΔAICc is the difference between the candidate model and the model having the lowest AICc, and ω_i_ is the AICc weight of each model. Models retained based on the lowest AICc value appears in bold, and models retained based on parsimony rules in italic font.

Model	Model family	Np	AICc	ΔAICc	ωi
A) Problem solving success	Binomial				
** ~ 1 + (1|Coyote ID)**	** **	**2**	**10.737**	**0.000**	**0.726**
~ Treatment group + (1|Coyote ID)		3	12.791	2.054	0.260
~ Trial number + (1|Coyote ID)		7	19.281	8.544	0.010
~ Treatment group + Trial number + (1|Coyote ID)		9	21.463	10.726	0.003
~ Treatment group x Trial number + (1|Coyote ID)		12	30.677	19.940	0.000
B) Neophobia	Gaussian				
~ 1 + (1|Coyote ID) + (1|Pair ID)	Log-transformed	2	364.341	3.147	0.095
*~ Treatment group + (1|Coyote ID) + (1|Pair ID)*	* *	*3*	*362*.*373*	*1*.*179*	*0*.*253*
~ Trial number + (1|Coyote ID) + (1|Pair ID)		7	365.606	4.412	0.050
~ Treatment group + Trial number + (1|Coyote ID) + (1|Pair ID)		9	363.493	2.299	0.145
~ Treatment group x Trial number + (1|Coyote ID) + (1|Pair ID)		12	363.194	0.000	0.457
C) Neophobia within the observer treatment group	Gaussian				
~ 1 + (1|Coyote ID) + (1|Pair ID) + (1|Age class)	Log-transformed	3	299.538	7.425	0.010
~ Sex + (1|Coyote ID) + (1|Pair ID) + (1|Age class)		5	298.815	6.702	0.014
*~ Social rank + (1|Coyote ID) + (1|Pair ID) + (1|Age class)*	* *	*6*	*292*.*136*	*0*.*023*	*0*.*402*
~ Sex + Social rank + (1|Coyote ID) + (1|Pair ID) + (1|Age class)		8	293.891	1.778	0.167
~ Sex * Social rank + (1|Coyote ID) + (1|Pair ID) + (1|Age class)		9	292.113	0.000	0.407
D) Persistence	Gaussian				
~ 1 + (1|Coyote ID) + (1|Pair ID)	Log-transformed	2	374.470	2.990	0.097
*~ Treatment group + (1|Coyote ID) + (1|Pair ID)*	* *	*3*	*372*.*597*	*1*.*117*	*0*.*248*
~ Trial number + (1|Coyote ID) + (1|Pair ID)		7	373.219	1.739	0.182
~ Treatment group + Trial number + (1|Coyote ID) + (1|Pair ID)		9	371.480	0.000	0.434
~ Treatment group x Trial number + (1|Coyote ID) + (1|Pair ID)		12	376.293	4.813	0.039
E) Persistence within the observer treatment group	Gaussian				
~ 1 + (1|Coyote ID) + (1|Pair ID) + (1|Age class)	Log-transformed	3	274.163	5.804	0.026
~ Sex + (1|Coyote ID) + (1|Pair ID) + (1|Age class)		5	271.519	3.160	0.097
** ~ Social rank + (1|Coyote ID) + (1|Pair ID) + (1|Age class)**	** **	**6**	**268.359**	**0.000**	**0.471**
~ Sex + Social rank + (1|Coyote ID) + (1|Pair ID) + (1|Age class)		8	270.085	1.726	0.199
~ Sex * Social rank + (1|Coyote ID) + (1|Pair ID) + (1|Age class)		9	270.007	1.648	0.207
F) Latency to eat inside the hoop	Gamma				
~ 1 + (1|Pair ID) + (1|Age class)		2	186.584	33.961	0.000
~ Treatment group + (1|Pair ID) + (1|Age class)		4	188.570	36.127	0.000
~ Sex + (1|Pair ID) + (1|Age class)		4	167.772	15.149	0.000
** ~ Social rank + (1|Pair ID) + (1|Age class)**	** **	**5**	**152.623**	**0.000**	**0.948**
~ Sex + Social rank + (1|Pair ID) + (1|Age class)		7	158.442	5.819	0.052
~ Sex * Social rank + (1|Pair ID) + (1|Age class)		8	211.037	58.414	0.000

**Fig 4:**
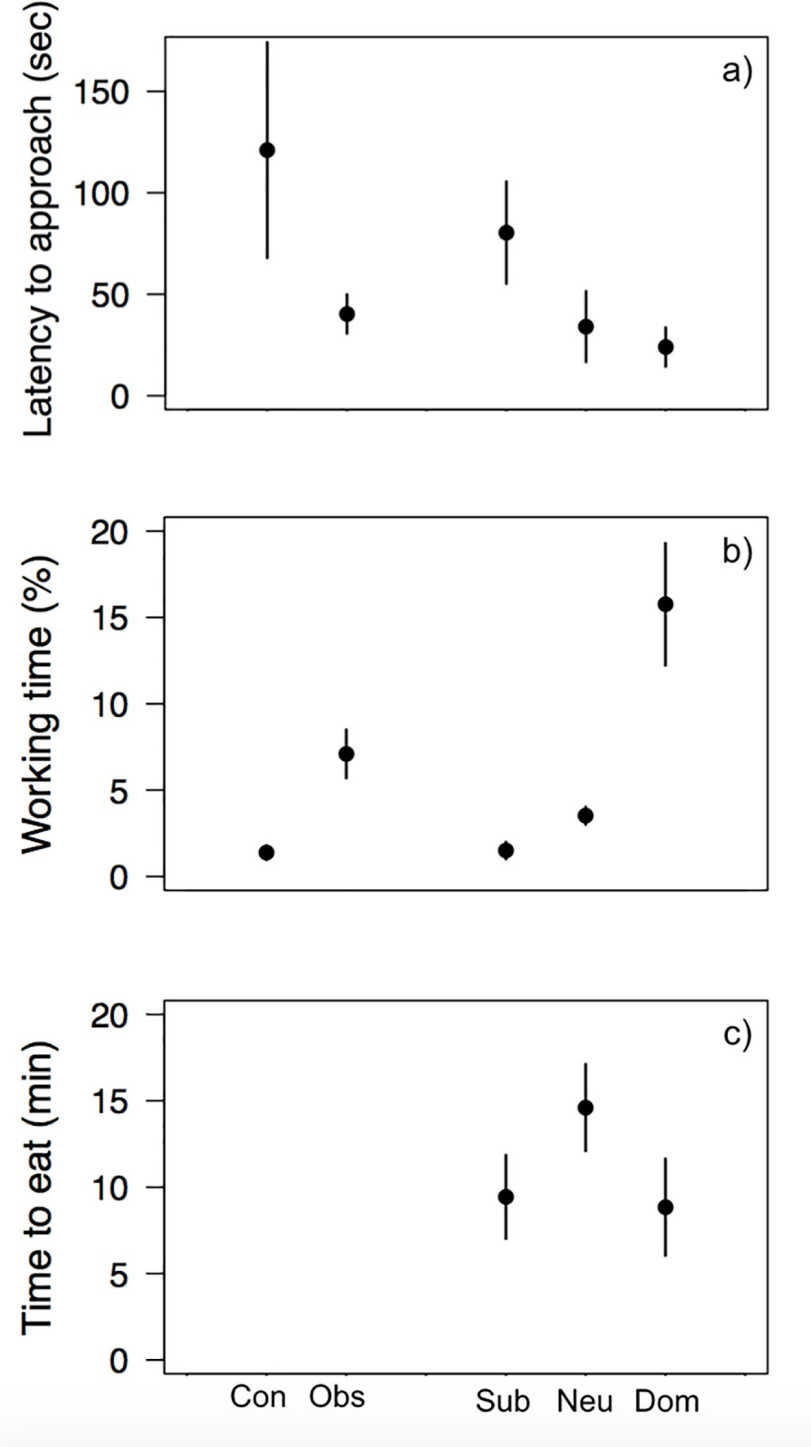
Mean time ± SE in Study 3 for control (Con) and observer (Obs) coyotes, and for subordinate (Sub), neutral (Neu) and dominant (Dom) individuals within the observer treatment group (a) required to approach the experimental apparatus (seconds) and (b) spent working on the problem. Mean time ± SE for subordinate, neutral and dominant unsuccessful individuals to eat the food inside the hoop during the post-trial tests (minutes).

There was a tendency for observer coyotes to be less neophobic than control individuals (Table 5B; β = -1.31 ± 0.73) and to spend more time on average working the apparatus (Table 5D; β = 0.78 ± 0.37). Observer coyotes approached the hoop on average 40.3 ± 9.6 seconds after the beginning of the trial, while it took 121.0 ± 53.1 seconds to control individuals (Fig 4A). Indeed, observer coyotes spent on average 7.1 ± 1.4% of the trial working on the problem, while control coyotes worked on the problem during only 1.4 ± 0.4% of the trial (Fig 4B). Similar tendencies within the observer treatment group were observed when individual characteristics were of interest.

Social rank was retained by all models as the best predictor of both neophobia and persistence within this treatment group (Table 5C and 5E). Subordinate individuals were found to be more neophobic than dominant individuals (β = 1.57 ± 0.51), and thus approached the experimental apparatus later (Fig 4A), while there was an unclear tendency of neutral individuals being slightly less neophobic than dominant individuals (β = -0.20 ± 0.50; Fig 4A). On the other hand, subordinate (β = -1.42 ± 0.40) and neutral (β = -0.35 ± 0.44) individuals were both found to be less persistent than dominant coyotes. Indeed, dominant individuals spent on average 15.8 ± 3.5% of the trial working on the problem, compared to 1.5 ± 0.5% and 3.5 ± 0.52% for subordinate and neutral individuals, respectively (Fig 4B).

Finally, all unsuccessful coyotes (i.e. observer and control coyotes of all tested pairs, n = 26 individuals) ate food from within the hoop during post-trial tests. Only social rank, and not treatment group and sex, was found to be a significant predictor of latency to eat the food from inside the hoop (Table 4F). Subordinate individuals required more time to eat inside the hoop than dominant individuals (β = 1.64 ± 0.24; Fig 4C). Neutral individuals also were slower than dominant individuals to eat inside the hoop, however the tendency was weak (β = 2.59 ± 4.87; Fig 4C). Finally, individual 0900 used in both studies 1 and 3 did not show any sign of habituation and responded the same way to the experimental design that individuals who had never been used in a social learning study before. Analyses conducted with and without this individual provided us with the same results.

## Discussion

This study adds to the growing body of literature illustrating social learning in wildlife. Our results suggest that coyotes can learn to perform a novel task through social learning and that persistent individuals will be more successful at solving novel tasks. We also observed that social rank (i.e., being a dominant male) likely matters for success; only dominant individuals within a male-female pair of socially bonded coyotes successfully solved tasks in studies 2 and 3. Our results were more ambiguous regarding the influence of object neophobia on social learning. While neophobia did not relate to social rank or vary significantly between control and observer coyotes in study 2, it may have influenced overall success rate as the proportion of total coyotes able to succeed in study 1, the only study without object neophobia, was much higher than in studies 2 or 3. Further, in study 2, latency to approach times decreased across trials, suggesting object neophobia may have initially occurred. Results from studies 1 and 2, that indicate greater success and more persistence among observers, are similar to those of other social learning studies that used an object manipulation task and a direct food reward [20,59]. In contrast, results from study 3 indicate relative to other taxa coyotes may perform more poorly at social-learning tasks where a food reward is decoupled from the apparatus [38–40].

The apparatus for study 1 was designed to be solvable by any coyote and this was evident because the task was successfully performed by both observer and control coyotes. We attempted to remove object neophobia entirely in study 1 by allowing all of the coyotes to have access to an open puzzle box leading up to testing trials. There was no relationship between trial number and success, latency to approach, or persistence, supporting the notion that object neophobia did not overtly influence coyote interactions with the puzzle box. Even though control and observer coyotes observed a conspecific eat from the puzzle box and were able to eat from the puzzle box before the problem-solving task was presented, differences between observer and control coyotes emerged that support the role of social learning in enhancing task performance. Coyotes which observed a conspecific solve a puzzle box to access a food reward during study 1 were significantly more successful, persistent, and faster to solve the task than coyotes that did not have a coyote demonstrate the task.

The apparatus used during studies 2 and 3 was also designed to be a task coyotes could perform, with the task in study 2 likely solvable by any coyote. Similar to study 1, coyotes were more attentive to and persistent with the testing apparatus during study 2 if they observed a demonstrator coyote. While at least the dominant individual (i.e., male in dyad) observer coyotes could solve the task in study 2, only two observer coyotes successfully performed the task during study 3 and no control coyotes successfully performed the tasks in studies 2 or 3.

Coyotes that solved the tasks in studies 1 and 2 once were consistently successful over subsequent trials. However, we did not see the improved rate of success over trials for the coyotes in study 1 that we observed in study 2. In study 1, we cannot eliminate the possibility that individual coyote performance, in terms of latency to solve, did not improve across trials because of a startle response to removing the door and was the main reason we altered the apparatus for studies 2 and 3.

Persistence is important when first interacting with a novel task because it allows an animal with little experience an opportunity to obtain a reward and may reduce the time to solve the task [59]. Observer coyotes showed significantly greater persistence toward solving the puzzle box than did control coyotes during study 1, the same trend was observed in successful observer coyotes during study 2, and there was a trend toward greater time interacting with the apparatus in study 3. We were unable to disentangle persistence and success because observer coyotes were both more persistent and more successful. This positive feedback loop is commonly observed across a wide range of taxa; individuals that persist longer are more likely to solve a novel problem via social and asocial learning [43,60,61]. While it is likely that observer coyotes persisted longer because of social learning, we are unable to determine if this occurs because observing a demonstrator indirectly reduced object neophobia or for other reasons. Observers simply may have been more persistent due to having a period of indirect exposure to the apparatus during demonstrations and therefore habituated to its presence. There were no differences between observer and control coyotes in direct measurements of object neophobia (i.e., latency to approach) during study 2 but observer coyotes had shorter latency to approach times in study 3. Thus, it is likely that observer coyotes were more persistent because of the combined effects of observing the object, task performance, and resulting reward. Future studies may want to consider exposing the control animals to indirect exposure of the device to tease these potential mechanisms apart.

Although neophobia may not inhibit problem solving in some cases [25,26,27], individuals that fail to engage with a novel object or novel food resource due to neophobia also fail to solve a novel task [23,59,62]. Neophobia typically results in avoidance of novel objects by coyotes when in familiar settings, whereas coyotes that have become habituated to objects will approach faster [47]. In study 1, prior experience with the testing apparatus likely emboldened both observer and control coyotes to engage with and thereby succeed at solving the puzzle box. It is unclear if prior experience with the testing apparatus in study 2 would have allowed for greater success in task performance. In study 2, there was no relationship between latency to approach and problem solving success, suggesting all coyotes exhibited a similar neophobic response to the apparatus. However, object neophobia appears to reduce with exposure as latency to approach times decreased over trial days and were shorter for observer coyotes in study 3. Observing a conspecific solve any task that results in a food reward should have decreased neophobic behavior in all three studies. In fact, conspecific demonstrators were shown to play a key role in social learning of new feeding cues in other species [63]. This suggests that a combination of direct experience with the object (study 1) or repeated exposure (study 2) in addition to the observation of a demonstrator (all studies) likely reduced object neophobia. Further, we note these findings related to neophobia are limited because we did not measure neophobia using a classic test of approach a novel object with food reward compared to approach a food reward (e.g., [64]). Coyotes used on this study approach food rapidly during daily feeding. Thus, we cautiously presume latency to approach times we observed were determined by each coyote’s willingness to approach the object itself relative to no latency to approach food alone.

Along with persistence and object neophobia, social status, age, sex, and the particular relationship between observer and demonstrator may also be important predictors of social learning [65]. We excluded issues of age by only testing adult coyotes and we excluded the potential role of demonstrator-observer relationships by ensuring none of the observers were socially related to their demonstrators. For example, in study 1 where individuals were tested, none of the demonstrator or observer coyotes were the mates of the demonstrators or control demonstrators. Learning from closely associated conspecifics could be advantageous because the observer experiences the same environment as the demonstrator [48]. In fact, the identity of the demonstrator may be a predictor of problem solving-success of observers [61,65]. At the facility, all captive coyotes experience the same environment even though they are housed as mated pairs. While they only interact with their mate directly, they often observe and interact with neighbors at adjacent enclosure boundaries. In this experiment, we were able to take advantage of this common scenario to measure social learning. However, we did not measure affiliation among demonstrators and observers prior to testing. Although we have not noted this behavior during casual observations, it is possible dominant individuals are more likely to interact with neighbors within the facility and this could partially explain why only dominant individuals performed tasks in studies 2 and 3.

Our results suggest social rank may relate to problem solving success in coyotes; but we could not separate rank from sex since most of the dominant individuals within our study animals were males. Even so, we found relationships between social rank/sex with both persistence and neophobia within the observer groups during studies 2 and 3. In coyotes, dominant individuals can limit access to food resources for subordinates [44] and it is possible that dominant coyotes in our study were limiting access to the apparatus by their subordinate mates because of the associated food reward at the apparatus during study 2. Although in study 3 the food was decoupled from the apparatus, the same trend in persistence and social rank was observed. In study 3, dominant coyotes were also faster at obtaining food within the hoop during post-trial testing. Although the captive coyotes behave similarly to wild coyotes [53], dominance for food in a dyad may not reflect relationships occurring in a complex social hierarchy [43,44]. However, social dominance did not guarantee success as some coyotes, including the majority in study 3, could not succeed at the task. More research on the relationship between social learning and task performance in coyotes in warranted, especially in relation to dyad behavioral patterns.

In contrast to results from studies 1 and 2, we found that most coyotes were unable to solve the task in study 3 through social learning. When decoupling the object from the food reward, observing a conspecific demonstrator perform the task increased persistence and decreased latency to approach (i.e., neophobia) by dominant individuals within dyads. In fact, no control coyotes interacted with the hoop apparatus by the final day of trials, while observer coyotes continued to interact with the apparatus. All observer and control coyotes were willing to eat from the apparatus after testing ended, showing object neophobia would not entirely explain earlier avoidance of the apparatus.

It is interesting that the two coyotes to learn the indirect food reward task were hand-reared coyotes, suggesting these coyotes may have been better able to cue on the human or human-based cue (i.e., clicker) as well as the conspecific demonstrator. Studies conducted on wolves and domestic dogs have shown that the key difference between dog and wolf behavior is the dog’s ability to look at the signals provided by humans [66–68]. Despite two hand-reared successes in study 3, four other hand-reared coyotes did not learn this task. The two successful coyotes showed more interest in humans than most coyotes within the captive facility, including among other hand-reared individuals. This was the only task in which a human was present during the entirety of the trial because of logistical constraints associated with providing the food rewards immediately upon successful completion of the task. However, captive coyotes housed at the NWRC are fed daily by humans from inside their enclosure and are, therefore, used to human presence and associate such presence with food. Further, all of the unsuccessful coyotes succeeded in approaching the hoop and eating food within it while the same human investigator was present in the enclosure. Although the human in our study did not provide any direct cues during observer trials, the clicker cues during demonstrator trials may have been sufficient for the two hand-reared and human-affiliated coyotes to cue on human demonstrators and solve the task. Results on social learning have previously shown differences between human-reared and mother-reared populations [69], and more research in this area is warranted.

Finally, modalities used by successful observer coyotes to perform the task in study 1 were independent of the modality displayed by their demonstrator. Social learning may occur through imitation and can be an efficient way to learn about new environments or tasks from conspecific innovators [3,5,6]. Our puzzle box design did not allow for the bidirectional control procedure necessary to test the specific mechanisms underlying the social learning we observed. However, our experimental set up and modality results suggest that coyotes are likely to gain social information through something other than imitation, such as stimulus enhancement [62,70].

Our study contributes to the basic foundation for understanding social learning in coyotes and the role of persistence, neophobia, and social rank/sex. We demonstrated that social learning occurs in coyotes and may shorten the time to succeed, likely due to an increase in persistence among observer coyotes. Social rank may determine success, reduce neophobia, and increase persistence but results are somewhat ambiguous due to differing results in studies 2 and 3. Further, most coyotes were unable to perform a task without a direct food reward. Captive populations provide an opportunity to examine social learning in a controlled setting, but further investigations are needed to fully understand the effects of social hierarchy, age, and social interaction on social learning.

## Supporting information

S1 Table Detailed information regarding individuals used in Study 1. The first two digits of the Coyote ID provide the year of birth.
(DOCX)

S2 Table Details of the captive coyotes used in study 2. The treatment group, sex, age class, social rank, and whether it was ever successful in solving the task is given for each captive coyote. The first two digits of Coyote ID are the year the coyote was born.
(DOCX)

S3 Table Details of the captive coyotes used in study 3. Each captive coyote is listed along with its treatment group, sex, and social rank, and whether it was ever successful in solving the task. The first two digits of Coyote ID are the year the coyote was born. Time to eat within hoop (min:sec) are until the coyote was willing to consume food inside the hoop after social learning trials were completed.
(DOCX)

S4 Table Raw data for study 1, using a puzzle box for on social learning in captive coyotes. (DOCX)

S5 Table Raw data for study 2; coyote pairs with direct food reward. (DOCX)

S6 Table Raw data for study 3; coyote pairs with decoupled food reward. (DOCX)

S7 Table Raw data from post-trial eating task during study 3. (DOCX)

S1 Fig Distribution of the three classes of social rank between sexes. All individuals used in both studies 2 and 3 were considered. Using a Chi-square test of independence, we found that sex and social rank were highly dependent, for individuals used in both studies (P = 0.0002 and P = 0.027, respectively for study 2 and study 3), males being more dominant than females.
(DOCX)
