# Supplementary material for: Persistence and conspecific observations improve problem-solving abilities of coyotes
Source: PLoS One. 2019 Jul 10;14(7):e0218778. doi: 10.1371/journal.pone.0218778 (PMC6619663; doi:10.1371/journal.pone.0218778)
Supplement: S1 Table — The first two digits of the Coyote ID provide the year of birth. (DOCX) [file pone.0218778.s001.docx]

**S1 Table.** Details of the captive coyotes used in study 1. The first two digits of the Coyote ID provide the year of birth.

| **Coyote ID** | **Treatment group** | **Sex** | **Success during first trial** |
| --- | --- | --- | --- |
| 06102 | Control | F | N |
| 08073 | Control | M | N |
| 0900 | Control | F | N |
| 0961 | Control | M | Y |
| 1073 | Control | M | N |
| 06065 | Observer | M | Y |
| 08100 | Observer | F | Y |
| 08120 | Observer | F | N |
| 0953 | Observer | M | Y |
| 1051 | Observer | M | N |
