# Supplementary material for: Persistence and conspecific observations improve problem-solving abilities of coyotes
Source: PLoS One. 2019 Jul 10;14(7):e0218778. doi: 10.1371/journal.pone.0218778 (PMC6619663; doi:10.1371/journal.pone.0218778)
Supplement: S2 Table — The treatment group, sex, age class, social rank, and whether it was ever successful in solving the task is given for each captive coyote. The first two digits of Coyote ID are the year the coyote was born. (DOCX) [file pone.0218778.s002.docx]

**S2 Table.** Details of the captive coyotes used in study 2. The treatment group, sex, age class, social rank, and whether it was ever successful in solving the task is given for each captive coyote. The first two digits of Coyote ID are the year the coyote was born.

| **Coyote ID** | **Treatment group^a^** | **Sex** | **Age class^b^** | **Social rank and score^c^** | **Success** |
| --- | --- | --- | --- | --- | --- |
| 1411 | Demonstrator | M |  | NA | NA |
| 0700 | Observer 1 | F | A | Subordinate - 0 | N |
| 0953 | Observer 1 | M | A | Dominant - 5 | Y |
| 1130 | Observer 2 | F | B | Subordinate - 1 | N |
| 1151 | Observer 2 | M | B | Dominant - 4 | Y |
| 1526 | Observer 3 | F | C | Subordinate - 0 | N |
| 1533 | Observer 3 | M | C | Dominant - 5 | N |
| 1252 | Observer 4 | F | A | Subordinate - 0 | N |
| 1211 | Observer 4 | M | A | Dominant - 5 | Y |
| 0957 | Demonstrator 2 | M |  | NA | NA |
| 1040 | Observer 1 | F | A | Dominant - 4 | N |
| 1033 | Observer 1 | M | A | Subordinate - 1 | N |
| 1032* | Observer 2 | F | B | Subordinate - 0 | N |
| 1231 | Observer 2 | M | B | Dominant - 5 | Y |
| 1406* | Observer 3 | F | C | Subordinate - 0 | N |
| 1417* | Observer 3 | M | C | Dominant - 5 | N |
| 1528 | Observer 4 | F | C | Subordinate - 1 | N |
| 1511 | Observer 4 | M | C | Dominant - 4 | N |
| 1060 | Control 1 | F | A | Subordinate - 0 | N |
| 1011 | Control 1 | M | A | Dominant - 5 | N |
| 1172 | Control 2 | F | B | Subordinate - 0 | N |
| 1071 | Control 2 | M | B | Dominant - 5 | N |
| 1510 | Control 3 | F | C | Subordinate - 1 | N |
| 1501 | Control 3 | M | C | Dominant - 4 | N |
| 1524 | Control 4 | F | C | Subordinate - 1 | N |
| 1535 | Control 4 | M | C | Dominant - 4 | N |

^a^Treatment group refers to whether the subject was a demonstrator, observer of the demonstrator 1411, observer of the demonstrator 0957, or in the control group that did not have access to a demonstrator.

^b^Class A refers to “old” adult coyotes ranging in age from 7 to 9, class B refers to adult coyotes ranging in age from 3 to 6, and class C refers to “young” adult coyotes ranging in age from 1 to 2.

^c^Social rank refers to the rank of the subject relative to the one other coyote with whom they were housed and the score (out of 5) obtained during the winner-loser trials for food dominance.

*individuals that were hand-reared.
