# Supplementary material for: Persistence and conspecific observations improve problem-solving abilities of coyotes
Source: PLoS One. 2019 Jul 10;14(7):e0218778. doi: 10.1371/journal.pone.0218778 (PMC6619663; doi:10.1371/journal.pone.0218778)
Supplement: S3 Table — Each captive coyote is listed along with its treatment group, sex, and social rank, and whether it was ever successful in solving the task. The first two digits of Coyote ID are the year the coyote was born. Time to eat within hoop (min:sec) are until the coyote was willing to consume food inside the hoop after social learning trials were completed. (DOCX) [file pone.0218778.s003.docx]

**S3 Table.** Details of the captive coyotes used in study 3. Each captive coyote is listed along with its treatment group, sex, and social rank, and whether it was ever successful in solving the task. The first two digits of Coyote ID are the year the coyote was born. Time to eat within hoop (min:sec) are until the coyote was willing to consume food inside the hoop after social learning trials were completed.

| **Coyote ID** | **Treatment group^a^** | **Sex** | **Age class^b^** | **Social rank and score^c^** | **Success** | **Time to eat within hoop** |
| --- | --- | --- | --- | --- | --- | --- |
| 1411 | Demonstrator 1 | M |  |  |  |  |
| 1054 | Observer 1 | F | A | Subordinate - 0 | N | 6:18 |
| 1031* | Observer 1 | M | A | Dominant - 5 | N | 6:02 |
| 1134 | Observer 2 | F | B | Subordinate - 0 | N | 5:32 |
| 1113 | Observer 2 | M | B | Dominant - 5 | N | 5:06 |
| 1408* | Observer 3 | F | C | Dominant - 4 | N | 1:36 |
| 1423 | Observer 3 | M | C | Subordinate - 1 | N | 2:01 |
| 1422 | Observer 4 | F | C | Subordinate - 1 | N | 4:36 |
| 1413* | Observer 4 | M | C | Dominant - 4 | Y | 0:33 |
| 1220 | Observer 5 | F | B | Neutral - 2 | N | 23:18 |
| 1201 | Observer 5 | M | B | Neutral - 3 | N | 23:17 |
| 1311 | Demonstrator 2 | M |  |  |  |  |
| 0920 | Observer 1 | F | A | Subordinate - 0 | N | 5:47 |
| 0951 | Observer 1 | M | A | Dominant - 5 | N | 5:08 |
| 1410* | Observer 2 | F | C | Subordinate - 0 | N | 2:46 |
| 1403* | Observer 2 | M | C | Dominant - 5 | Y | 0:22 |
| 1210 | Observer 3 | F | B | Subordinate - 1 | N | 17:56 |
| 1251 | Observer 3 | M | B | Dominant - 4 | N | 19:32 |
| 1230 | Observer 4 | F | B | Neutral - 3 | N | 9:19 |
| 1241 | Observer 4 | M | B | Neutral - 2 | N | 10:15 |
| 1070 | Observer 5 | F | A | Neutral - 2 | N | 18:46 |
| 1033* | Observer 5 | M | A | Neutral - 3 | N | 19:11 |
| 0900 | Control 1 | F | A | Subordinate - 0 | N | 9:12 |
| 08063 | Control 1 | M | A | Dominant - 1 | N | 8:34 |
| 0950 | Control 2 | F | B | Neutral - 2 | N | 7:02 |
| 1141 | Control 2 | M | B | Neutral - 3 | N | 6:55 |
| 1400* | Control 3 | F | C | Subordinate - 1 | N | 17:32 |
| 1421 | Control 3 | M | C | Dominant - 4 | N | 16:49 |
| 1162 | Control 4 | F | B | Subordinate - 0 | N | 24:54 |
| 1143 | Control 4 | M | B | Dominant - 1 | N | 26:18 |

^a^Classification of each coyote as a demonstrator, observer of the demonstrator 1411, observer of the demonstrator 1311, or in the control group that did not have access to a demonstrator.

^b^Class A refers to “old” adult coyotes ranging in age from 7 to 9, class B refers to adult coyotes ranging in age from 3 to 6, and class C refers to “young” adult coyotes ranging in age from 1 to 2.

^c^The rank of the subject relative to the one other coyote with whom they were housed and the score (out of 5) obtained during the winner-loser trials for food dominance.

*Individuals that were hand reared
