# Supplementary material for: Persistence and conspecific observations improve problem-solving abilities of coyotes
Source: PLoS One. 2019 Jul 10;14(7):e0218778. doi: 10.1371/journal.pone.0218778 (PMC6619663; doi:10.1371/journal.pone.0218778)
Supplement: S4 Table — (DOCX) [file pone.0218778.s004.docx]

| **S4 Table.** Raw data for study 1, using a puzzle box for on social learning in captive coyotes. | | | | | | | | | |
| --- | --- | --- | --- | --- | --- | --- | --- | --- | --- |
| **Coyote ID** | **Sex** | **Treatment** | **Test Day** | **Door pulled off** | **Successful** | **% Time WI2** | **% Time EN** | **Latency to solve** | **Latency to pull the door** |
| 06065 | M | Student | 1 | Y | Y | 0.89 | 0.41 | 430 | 82 |
| 08073 | M | Control | 1 | Y | N | 0.56 | 0.19 | NA | NA |
| 08100 | F | Student | 1 | Y | Y | 0.64 | 0.20 | 577 | 355 |
| 08120 | F | Student | 1 | N | N | 0.15 | 0.01 | NA | NA |
| 0900 | F | Control | 1 | N | N | 0.06 | 0.01 | NA | NA |
| 0953 | M | Student | 1 | Y | Y | 0.98 | 0.77 | 95 | 65 |
| 0961 | M | Control | 1 | Y | Y | 0.17 | 0.07 | 5750 | 5721 |
| 1051 | M | Student | 1 | Y | Y | 0.35 | 0.10 | 2738 | 1476 |
| 1073 | M | Control | 1 | Y | N | 0.14 | 0.07 | NA | NA |
| 06065 | M | Student | 2 | Y | Y | 0.76 | 0.26 | 178 | 96 |
| 06102 | F | Control | 2 | N | N | 0.09 | 0.03 | NA | NA |
| 08073 | M | Control | 2 | N | N | 0.10 | 0.02 | NA | NA |
| 08100 | F | Student | 2 | Y | Y | 0.67 | 0.15 | 1031 | 920 |
| 08120 | F | Student | 2 | Y | Y | 0.90 | 0.20 | 1478 | 684 |
| 0900 | F | Control | 2 | N | N | 0.06 | 0.10 | NA | NA |
| 0953 | M | Student | 2 | Y | Y | 0.99 | 0.63 | 69 | 46 |
| 0961 | M | Control | 2 | Y | Y | 0.44 | 0.11 | 6410 | 6286 |
| 1051 | M | Student | 2 | Y | Y | 0.62 | 0.20 | 1584 | 993 |
| 1073 | M | Control | 2 | N | N | 0.03 | 0.01 | NA | NA |
| 06065 | M | Student | 3 | Y | Y | 0.89 | 0.44 | 784 | 180 |
| 06102 | F | Control | 3 | N | N | 0.06 | 0.03 | NA | NA |
| 08073 | M | Control | 3 | Y | Y | 0.50 | 0.21 | 6726 | 4913 |
| 08100 | F | Student | 3 | Y | Y | 0.52 | 0.13 | 1611 | 1580 |
| 08120 | F | Student | 3 | Y | Y | 0.78 | 0.28 | 1725 | 1623 |
| 0900 | F | Control | 3 | N | N | 0.01 | 0.00 | NA | NA |
| 0953 | M | Student | 3 | Y | Y | 0.85 | 0.60 | 46.00 | 35 |
| 0961 | M | Control | 3 | N | N | 0.16 | 0.01 | NA | NA |
| 1051 | M | Student | 3 | Y | Y | 0.32 | 0.12 | 2073 | 1998 |
| 1073 | M | Control | 3 | N | N | 0.01 | 0.01 | NA | NA |
| 06065 | M | Student | 4 | Y | Y | 0.69 | 0.22 | 709 | 474 |
| 06102 | F | Control | 4 | N | N | 0.04 | 0.01 | NA | NA |
| 08073 | M | Control | 4 | N | N | 0.07 | 0.02 | NA | NA |
| 08100 | F | Student | 4 | Y | Y | 0.45 | 0.04 | 1914 | 1838 |
| 08120 | F | Student | 4 | N | N | 0.15 | 0.02 | NA | NA |
| 0900 | F | Control | 4 | N | N | 0.02 | 0.00 | NA | NA |
| 0953 | M | Student | 4 | Y | Y | 0.82 | 0.41 | 46.00 | 33 |
| 0961 | M | Control | 4 | N | N | 0.03 | 0.00 | NA | NA |
| 1051 | M | Student | 4 | Y | Y | 0.44 | 0.16 | 1307 | 1271 |
| 1073 | M | Control | 4 | Y | Y | 0.50 | 0.24 | 979 | 737 |
| 06065 | M | Student | 5 | Y | Y | 0.90 | 0.30 | 657 | 373 |
| 06102 | F | Control | 5 | Y | Y | 0.16 | 0.05 | 2714 | 2387 |
| 08073 | M | Control | 5 | Y | N | 0.35 | 0.11 | NA | NA |
| 08100 | F | Student | 5 | Y | Y | 0.54 | 0.07 | 1544 | 1473 |
| 08120 | F | Student | 5 | Y | Y | 0.76 | 0.33 | 1925 | 629 |
| 0900 | F | Control | 5 | N | N | 0.01 | 0.00 | NA | NA |
| 0953 | M | Student | 5 | Y | Y | 0.50 | 0.22 | 39.00 | 27 |
| 0961 | M | Control | 5 | Y | Y | 0.07 | 0.01 | 5328 | 5260 |
| 1051 | M | Student | 5 | Y | Y | 0.22 | 0.06 | 2234 | 2234 |
| 1073 | M | Control | 5 | Y | Y | 0.51 | 0.21 | 1940 | 1659 |
