# Supplementary material for: Persistence and conspecific observations improve problem-solving abilities of coyotes
Source: PLoS One. 2019 Jul 10;14(7):e0218778. doi: 10.1371/journal.pone.0218778 (PMC6619663; doi:10.1371/journal.pone.0218778)
Supplement: S5 Table — (DOCX) [file pone.0218778.s005.docx]

| **S5 Table.** Raw data for study 2; coyote pairs with direct food reward. | | | | | | | | | | | | | |
| --- | --- | --- | --- | --- | --- | --- | --- | --- | --- | --- | --- | --- | --- |
| **ID** | **Demo ID** | **Demo modality** | **Treat Group** | **Test Day** | **Rearing** | **Social Rank** | **% of time toward the demo** | **Success** | **Latency to approach** | **Student Modality** | **Latency to solve** | **% of working time** | **Interaction** |
| 0700 | 1411 | Paw | Observer | 1 | NO | Subordinate | 78.12 | N | 2 | NA | NA | 32.18 | Competition |
| 0953 | 1411 | Paw | Observer | 1 | NO | Dominant | 81.36 | Y | 0 | Paw | 134 | 100.00 | Competition |
| 1060 | NO DEM | NA | Control | 1 | NO | Subordinate | 0.00 | N | 46 | NA | NA | 5.26 | Facilitation |
| 1011 | NO DEM | NA | Control | 1 | NO | Dominant | 0.00 | N | 32 | NA | NA | 12.55 | Facilitation |
| 0700 | 1411 | Paw | Observer | 2 | NO | Subordinate | 82.17 | N | 18 | NA | NA | 27.18 | Competition |
| 0953 | 1411 | Paw | Observer | 2 | NO | Dominant | 85.24 | Y | 0 | Paw | 122 | 100.00 | Competition |
| 1060 | NO DEM | NA | Control | 2 | NO | Subordinate | 0.00 | N | 54 | NA | NA | 4.35 | Facilitation |
| 1011 | NO DEM | NA | Control | 2 | NO | Dominant | 0.00 | N | 12 | NA | NA | 10.12 | Facilitation |
| 0700 | 1411 | Paw | Observer | 3 | NO | Subordinate | 75.25 | N | 21 | NA | NA | 25.76 | Competition |
| 0953 | 1411 | Paw | Observer | 3 | NO | Dominant | 81.75 | Y | 0 | Paw | 89 | 100.00 | Competition |
| 1060 | NO DEM | NA | Control | 3 | NO | Subordinate | 0.00 | N | 47 | NA | NA | 3.94 | Facilitation |
| 1011 | NO DEM | NA | Control | 3 | NO | Dominant | 0.00 | N | 11 | NA | NA | 8.55 | Facilitation |
| 0700 | 1411 | Paw | Observer | 4 | NO | Subordinate | 67.36 | N | 7 | NA | NA | 19.43 | Competition |
| 0953 | 1411 | Paw | Observer | 4 | NO | Dominant | 93.56 | Y | 0 | Paw | 65 | 100.00 | Competition |
| 1060 | NO DEM | NA | Control | 4 | NO | Subordinate | 0.00 | N | 51 | NA | NA | 2.33 | Facilitation |
| 1011 | NO DEM | NA | Control | 4 | NO | Dominant | 0.00 | N | 27 | NA | NA | 7.13 | Facilitation |
| 0700 | 1411 | Paw | Observer | 5 | NO | Subordinate | 71.28 | N | 7 | NA | NA | 18.16 | Competition |
| 0953 | 1411 | Paw | Observer | 5 | NO | Dominant | 82.33 | Y | 0 | Paw | 52 | 100.00 | Competition |
| 1060 | NO DEM | NA | Control | 5 | NO | Subordinate | 0.00 | N | 43 | NA | NA | 1.12 | Facilitation |
| 1011 | NO DEM | NA | Control | 5 | NO | Dominant | 0.00 | N | 37 | NA | NA | 5.26 | Facilitation |
| 1130 | 1411 | Paw | Observer | 1 | NO | Subordinate | 51.18 | N | 12 | NA | NA | 22.18 | Facilitation |
| 1151 | 1411 | Paw | Observer | 1 | NO | Dominant | 62.33 | N | 0 | NA | NA | 45.74 | Facilitation |
| 1172 | NO DEM | NA | Control | 1 | NO | Subordinate | 0.00 | N | 8 | NA | NA | 18.26 | Competition |
| 1071 | NO DEM | NA | Control | 1 | NO | Dominant | 0.00 | N | 2 | NA | NA | 51.76 | Competition |
| 1032 | 0953 | Muzzle | Observer | 1 | YES | Subordinate | 45.26 | N | 21 | NA | NA | 11.28 | Cooperation |
| 1231 | 0953 | Muzzle | Observer | 1 | NO | Dominant | 57.89 | Y | 13 | Paw | 389 | 78.12 | Cooperation |
| 1130 | 1411 | Paw | Observer | 2 | NO | Subordinate | 45.78 | N | 15 | NA | NA | 19.26 | Competition |
| 1151 | 1411 | Paw | Observer | 2 | NO | Dominant | 59.33 | Y | 2 | Paw | 618 | 58.33 | Competition |
| 1172 | NO DEM | NA | Control | 2 | NO | Subordinate | 0.00 | N | 10 | NA | NA | 20.22 | Competition |
| 1071 | NO DEM | NA | Control | 2 | NO | Dominant | 0.00 | N | 0 | NA | NA | 47.32 | Competition |
| 1032 | 0953 | Muzzle | Observer | 2 | YES | Subordinate | 44.12 | N | 15 | NA | NA | 17.28 | Cooperation |
| 1231 | 0953 | Muzzle | Observer | 2 | NO | Dominant | 55.33 | Y | 7 | Paw | 256 | 85.65 | Cooperation |
| 1130 | 1411 | Paw | Observer | 3 | NO | Subordinate | 43.26 | N | 20 | NA | NA | 12.10 | Competition |
| 1151 | 1411 | Paw | Observer | 3 | NO | Dominant | 55.18 | Y | 0 | Paw | 575 | 45.18 | Competition |
| 1172 | NO DEM | NA | Control | 3 | NO | Subordinate | 0.00 | N | 8 | NA | NA | 11.33 | Cooperation |
| 1071 | NO DEM | NA | Control | 3 | NO | Dominant | 0.00 | N | 0 | NA | NA | 55.33 | Competition |
| 1032 | 0953 | Muzzle | Observer | 3 | YES | Subordinate | 43.12 | N | 10 | NA | NA | 19.56 | Cooperation |
| 1231 | 0953 | Muzzle | Observer | 3 | NO | Dominant | 50.78 | Y | 8 | Paw | 245 | 88.13 | Cooperation |
| 1130 | 1411 | Paw | Observer | 4 | NO | Subordinate | 41.72 | N | 18 | NA | NA | 8.33 | Competition |
| 1151 | 1411 | Paw | Observer | 4 | NO | Dominant | 52.73 | Y | 0 | Paw | 412 | 66.91 | Competition |
| 1172 | NO DEM | NA | Control | 4 | NO | Subordinate | 0.00 | N | 2 | NA | NA | 6.27 | Competition |
| 1071 | NO DEM | NA | Control | 4 | NO | Dominant | 0.00 | N | 0 | NA | NA | 46.13 | Competition |
| 1032 | 0953 | Muzzle | Observer | 4 | YES | Subordinate | 41.66 | N | 16 | NA | NA | 10.25 | Cooperation |
| 1231 | 0953 | Muzzle | Observer | 4 | NO | Dominant | 48.81 | Y | 9 | Paw | 57 | 99.33 | Cooperation |
| 1130 | 1411 | Paw | Observer | 5 | NO | Subordinate | 40.33 | N | 17 | NA | NA | 5.26 | Competition |
| 1151 | 1411 | Paw | Observer | 5 | NO | Dominant | 50.65 | Y | 2 | Paw | 212 | 72.18 | Competition |
| 1172 | NO DEM | NA | Control | 5 | NO | Subordinate | 0.00 | N | 10 | NA | NA | 4.22 | Competition |
| 1071 | NO DEM | NA | Control | 5 | NO | Dominant | 0.00 | N | 4 | NA | NA | 42.28 | Competition |
| 1032 | 0953 | Muzzle | Observer | 5 | YES | Subordinate | 38.51 | N | 9 | NA | NA | 5.16 | Cooperation |
| 1231 | 0953 | Muzzle | Observer | 5 | NO | Dominant | 47.18 | Y | 0 | Paw | 55 | 100.00 | Cooperation |
| 1406 | 0953 | Muzzle | Observer | 1 | YES | Subordinate | 51.72 | N | 80 | NA | NA | 22.06 | No Interaction |
| 1417 | 0953 | Muzzle | Observer | 1 | YES | Dominant | 66.18 | N | NA | NA | NA | 0.00 | No Interaction |
| 1524 | NO DEM | NA | Control | 1 | NO | Subordinate | 0.00 | N | NA | NA | NA | 0.00 | No Interaction |
| 1535 | NO DEM | NA | Control | 1 | NO | Dominant | 0.00 | N | NA | NA | NA | 0.00 | No Interaction |
| 1406 | 0953 | Muzzle | Observer | 2 | YES | Subordinate | 71.26 | N | 36 | NA | NA | 31.12 | No Interaction |
| 1417 | 0953 | Muzzle | Observer | 2 | YES | Dominant | 72.55 | N | NA | NA | NA | 0.00 | No Interaction |
| 1524 | NO DEM | NA | Control | 2 | NO | Subordinate | 0.00 | N | NA | NA | NA | 0.00 | No Interaction |
| 1535 | NO DEM | NA | Control | 2 | NO | Dominant | 0.00 | N | NA | NA | NA | 0.00 | No Interaction |
| 1406 | 0953 | Muzzle | Observer | 3 | YES | Subordinate | 62.36 | N | 22 | NA | NA | 45.78 | No Interaction |
| 1417 | 0953 | Muzzle | Observer | 3 | YES | Dominant | 64.28 | N | NA | NA | NA | 0.00 | No Interaction |
| 1524 | NO DEM | NA | Control | 3 | NO | Subordinate | 0.00 | N | NA | NA | NA | 0.00 | No Interaction |
| 1535 | NO DEM | NA | Control | 3 | NO | Dominant | 0.00 | N | NA | NA | NA | 0.00 | No Interaction |
| 1406 | 0953 | Muzzle | Observer | 4 | YES | Subordinate | 51.19 | N | 12 | NA | NA | 50.33 | No Interaction |
| 1417 | 0953 | Muzzle | Observer | 4 | YES | Dominant | 62.36 | N | NA | NA | NA | 0.00 | No Interaction |
| 1524 | NO DEM | NA | Control | 4 | NO | Subordinate | 0.00 | N | NA | NA | NA | 0.00 | No Interaction |
| 1535 | NO DEM | NA | Control | 4 | NO | Dominant | 0.00 | N | NA | NA | NA | 0.00 | No Interaction |
| 1406 | 0953 | Muzzle | Observer | 5 | YES | Subordinate | 37.33 | N | 2 | NA | NA | 56.67 | No Interaction |
| 1417 | 0953 | Muzzle | Observer | 5 | YES | Dominant | 40.65 | N | NA | NA | NA | 0.00 | No Interaction |
| 1524 | NO DEM | NA | Control | 5 | NO | Subordinate | 0.00 | N | NA | NA | NA | 0.00 | No Interaction |
| 1535 | NO DEM | NA | Control | 5 | NO | Dominant | 0.00 | N | NA | NA | NA | 0.00 | No Interaction |
| 1510 | NO DEM | NA | Control | 1 | NO | Subordinate | 0.00 | N | 128 | NA | NA | 33.12 | No Interaction |
| 1501 | NO DEM | NA | Control | 1 | NO | Dominant | 0.00 | N | NA | NA | NA | 0.00 | No Interaction |
| 1040 | 0953 | Muzzle | Control | 1 | NO | Dominant | 0.00 | N | 26 | NA | NA | 25.26 | Cooperation |
| 1073 | 0953 | Muzzle | Control | 1 | NO | Subordinate | 0.00 | N | 8 | NA | NA | 29.67 | Cooperation |
| 1252 | 1411 | Paw | Observer | 1 | NO | Subordinate | 62.31 | N | 54 | NA | NA | 11.26 | Facilitation |
| 1211 | 1411 | Paw | Observer | 1 | NO | Dominant | 65.78 | N | 22 | NA | NA | 45.18 | Facilitation |
| 1526 | 1411 | Paw | Observer | 1 | NO | Subordinate | 76.54 | N | 78 | NA | NA | 33.69 | No Interaction |
| 1533 | 1411 | Paw | Observer | 1 | NO | Dominant | 78.22 | N | 102 | NA | NA | 2.18 | No Interaction |
| 1511 | 0953 | Muzzle | Observer | 1 | NO | Subordinate | 68.33 | N | 116 | NA | NA | 21.18 | No Interaction |
| 1528 | 0953 | Muzzle | Observer | 1 | NO | Dominant | 71.67 | N | 201 | NA | NA | 1.33 | No Interaction |
| 1510 | NO DEM | NA | Control | 2 | NO | Subordinate | 0.00 | N | 312 | NA | NA | 27.18 | No Interaction |
| 1501 | NO DEM | NA | Control | 2 | NO | Dominant | 0.00 | N | NA | NA | NA | 0.00 | No Interaction |
| 1040 | 0953 | Muzzle | Control | 2 | NO | Dominant | 0.00 | N | 12 | NA | NA | 31.33 | Cooperation |
| 1073 | 0953 | Muzzle | Control | 2 | NO | Subordinate | 0.00 | N | 4 | NA | NA | 34.67 | Cooperation |
| 1252 | 1411 | Paw | Observer | 2 | NO | Subordinate | 60.02 | N | 32 | NA | NA | 21.29 | Facilitation |
| 1211 | 1411 | Paw | Observer | 2 | NO | Dominant | 57.28 | Y | 8 | Muzzle | 956 | 57.33 | Facilitation |
| 1526 | 1411 | Paw | Observer | 2 | NO | Subordinate | 75.13 | N | 24 | NA | NA | 54.22 | No Interaction |
| 1533 | 1411 | Paw | Observer | 2 | NO | Dominant | 77.56 | N | 116 | NA | NA | 1.33 | No Interaction |
| 1511 | 0953 | Muzzle | Observer | 2 | NO | Subordinate | 69.16 | N | 98 | NA | NA | 33.56 | No Interaction |
| 1528 | 0953 | Muzzle | Observer | 2 | NO | Dominant | 68.25 | N | 110 | NA | NA | 0.67 | No Interaction |
| 1510 | NO DEM | NA | Control | 3 | NO | Subordinate | 0.00 | N | 256 | NA | NA | 37.12 | No Interaction |
| 1501 | NO DEM | NA | Control | 3 | NO | Dominant | 0.00 | N | 454 | NA | NA | 1.33 | No Interaction |
| 1040 | 0953 | Muzzle | Control | 3 | NO | Dominant | 0.00 | N | 4 | NA | NA | 18.99 | Cooperation |
| 1073 | 0953 | Muzzle | Control | 3 | NO | Subordinate | 0.00 | N | 0 | NA | NA | 21.72 | Cooperation |
| 1252 | 1411 | Paw | Observer | 3 | NO | Subordinate | 54.25 | N | 24 | NA | NA | 15.26 | Facilitation |
| 1211 | 1411 | Paw | Observer | 3 | NO | Dominant | 57.33 | Y | 0 | Muzzle | 734 | 61.18 | Facilitation |
| 1526 | 1411 | Paw | Observer | 3 | NO | Subordinate | 72.65 | N | 0 | NA | NA | 66.78 | No Interaction |
| 1533 | 1411 | Paw | Observer | 3 | NO | Dominant | 81.28 | N | NA | NA | NA | 0.00 | No Interaction |
| 1511 | 0953 | Muzzle | Observer | 3 | NO | Subordinate | 67.26 | N | 54 | NA | NA | 56.78 | No Interaction |
| 1528 | 0953 | Muzzle | Observer | 3 | NO | Dominant | 55.19 | N | 312 | NA | NA | 0.17 | No Interaction |
| 1510 | NO DEM | NA | Control | 4 | NO | Subordinate | 0.00 | N | 39 | NA | NA | 47.29 | No Interaction |
| 1501 | NO DEM | NA | Control | 4 | NO | Dominant | 0.00 | N | 237 | NA | NA | 0.67 | No Interaction |
| 1040 | 0953 | Muzzle | Control | 4 | NO | Dominant | 0.00 | N | 2 | NA | NA | 15.26 | Cooperation |
| 1073 | 0953 | Muzzle | Control | 4 | NO | Subordinate | 0.00 | N | 0 | NA | NA | 18.56 | Cooperation |
| 1252 | 1411 | Paw | Observer | 4 | NO | Subordinate | 51.33 | N | 22 | NA | NA | 16.78 | Facilitation |
| 1211 | 1411 | Paw | Observer | 4 | NO | Dominant | 54.67 | Y | 0 | Muzzle | 722 | 62.33 | Facilitation |
| 1526 | 1411 | Paw | Observer | 4 | NO | Subordinate | 68.59 | N | 0 | NA | NA | 65.33 | No Interaction |
| 1533 | 1411 | Paw | Observer | 4 | NO | Dominant | 71.25 | N | NA | NA | NA | 0.00 | No Interaction |
| 1511 | 0953 | Muzzle | Observer | 4 | NO | Subordinate | 89.17 | N | 12 | NA | NA | 62.18 | No Interaction |
| 1528 | 0953 | Muzzle | Observer | 4 | NO | Dominant | 76.58 | N | NA | NA | NA | 0.00 | No Interaction |
| 1510 | NO DEM | NA | Control | 5 | NO | Subordinate | 0.00 | N | 12 | NA | NA | 55.67 | No Interaction |
| 1501 | NO DEM | NA | Control | 5 | NO | Dominant | 0.00 | N | 556 | NA | NA | 0.33 | No Interaction |
| 1040 | 0953 | Muzzle | Control | 5 | NO | Dominant | 0.00 | N | 0 | NA | NA | 9.67 | Cooperation |
| 1073 | 0953 | Muzzle | Control | 5 | NO | Subordinate | 0.00 | N | 0 | NA | NA | 11.25 | Cooperation |
| 1252 | 1411 | Paw | Observer | 5 | NO | Subordinate | 62.31 | N | 9 | NA | NA | 20.18 | Facilitation |
| 1211 | 1411 | Paw | Observer | 5 | NO | Dominant | 65.78 | Y | 0 | Muzzle | 655 | 65.46 | Facilitation |
| 1526 | 1411 | Paw | Observer | 5 | NO | Subordinate | 76.54 | N | 2 | NA | NA | 71.26 | No Interaction |
| 1533 | 1411 | Paw | Observer | 5 | NO | Dominant | 78.22 | N | NA | NA | NA | 0.00 | No Interaction |
| 1511 | 0953 | Muzzle | Observer | 5 | NO | Subordinate | 68.33 | N | 0 | NA | NA | 65.78 | No Interaction |
| 1528 | 0953 | Muzzle | Observer | 5 | NO | Dominant | 71.67 | N | NA | NA | NA | 0.00 | No Interaction |
