# Supplementary material for: Persistence and conspecific observations improve problem-solving abilities of coyotes
Source: PLoS One. 2019 Jul 10;14(7):e0218778. doi: 10.1371/journal.pone.0218778 (PMC6619663; doi:10.1371/journal.pone.0218778)
Supplement: S6 Table — (DOCX) [file pone.0218778.s006.docx]

| **S6 Table.** Raw data for study 3; coyote pairs with decoupled food reward. | | | | | | | | | | | | |
| --- | --- | --- | --- | --- | --- | --- | --- | --- | --- | --- | --- | --- |
| **Coyote ID** | **Demo ID** | **Treat Group** | **Rearing** | **Social Rank** | **Trial number** | **% of time toward the demo** | **Number of times watching the performance** | **Latency to approach** | **Success** | **Latency to solve** | **% of working time** | **Pair interaction** |
| 0900 | NO DEM | Control | NO | Subordinate | 1 | 0 | 0 | NA | N | NA | 0 | No Interaction |
| 08063 | NO DEM | Control | NO | Dominant | 1 | 0 | 0 | NA | N | NA | 0 | No Interaction |
| 0950 | NO DEM | Control | NO | Neutral | 1 | 0 | 0 | 138 | N | NA | 6.08 | Facilitation |
| 1141 | NO DEM | Control | NO | Neutral | 1 | 0 | 0 | 80 | N | NA | 6.67 | Facilitation |
| 1400 | NO DEM | Control | YES | Subordinate | 1 | 0 | 0 | NA | N | NA | 0 | No Interaction |
| 1421 | NO DEM | Control | NO | Dominant | 1 | 0 | 0 | NA | N | NA | 0 | No Interaction |
| 1162 | NO DEM | Control | NO | Dominant | 1 | 0 | 0 | 0 | N | NA | 1.17 | Facilitation |
| 1143 | NO DEM | Control | NO | Subordinate | 1 | 0 | 0 | 155 | N | NA | 1.33 | Facilitation |
| 1054 | 1411 | Observer | NO | Subordinate | 1 | 25.33 | 1 | NA | N | NA | 0 | No Interaction |
| 1031 | 1411 | Observer | YES | Dominant | 1 | 49 | 2 | 0 | N | NA | 2.33 | No Interaction |
| 1134 | 1411 | Observer | NO | Subordinate | 1 | 53.33 | 3 | 26 | N | NA | 2.17 | Facilitation |
| 1113 | 1411 | Observer | NO | Dominant | 1 | 48.52 | 3 | 8 | N | NA | 4.25 | Facilitation |
| 0920 | 1311 | Observer | NO | Subordinate | 1 | 19.88 | 3 | 10 | N | NA | 1.25 | Cooperation |
| 0951 | 1311 | Observer | NO | Dominant | 1 | 19.88 | 3 | 6 | N | NA | 7.00 | Cooperation |
| 1408 | 1411 | Observer | YES | Dominant | 1 | 39.05 | 1 | 12 | N | NA | 14.75 | No Interaction |
| 1423 | 1411 | Observer | NO | Subordinate | 1 | 85.24 | 3 | 0 | N | NA | 14.83 | No Interaction |
| 1410 | 1311 | Observer | YES | Subordinate | 1 | 38.95 | 3 | NA | N | NA | 0 | No Interaction |
| 1403 | 1311 | Observer | YES | Dominant | 1 | 73.16 | 3 | 0 | Y | 738 | 16.66 | No Interaction |
| 1230 | 1311 | Observer | NO | Neutral | 1 | 38.82 | 3 | 15 | N | NA | 2.58 | Cooperation |
| 1241 | 1311 | Observer | NO | Neutral | 1 | 19.21 | 3 | 19 | N | NA | 8.25 | Cooperation |
| 1422 | 1411 | Observer | NO | Subordinate | 1 | 75.83 | 3 | NA | N | NA | 0 | No Interaction |
| 1413 | 1411 | Observer | YES | Dominant | 1 | 59.58 | 3 | 0 | Y | 252 | 53.72 | No Interaction |
| 1220 | 1411 | Observer | NO | Neutral | 1 | 39.05 | 2 | 80 | N | NA | 0.51 | No Interaction |
| 1201 | 1411 | Observer | NO | Neutral | 1 | 70.95 | 3 | NA | N | NA | 0.00 | No Interaction |
| 1210 | 1311 | Observer | NO | Subordinate | 1 | 72.22 | 3 | NA | N | NA | 0.00 | No Interaction |
| 1251 | 1311 | Observer | NO | Dominant | 1 | 68.52 | 3 | 0 | N | NA | 9.17 | No Interaction |
| 1070 | 1311 | Observer | NO | Neutral | 1 | 39.02 | 3 | 18 | N | NA | 5.75 | Competition |
| 1033 | 1311 | Observer | YES | Neutral | 1 | 50.39 | 3 | 4 | N | NA | 5.00 | Competition |
| 0900 | NO DEM | Control | NO | Subordinate | 2 | 0 | 0 | 368 | N | NA | 0.58 | No Interaction |
| 08063 | NO DEM | Control | NO | Dominant | 2 | 0 | 0 | NA | N | NA | 0 | No Interaction |
| 0950 | NO DEM | Control | NO | Neutral | 2 | 0 | 0 | 72 | N | NA | 5.17 | Facilitation |
| 1141 | NO DEM | Control | NO | Neutral | 2 | 0 | 0 | 0 | N | NA | 5.92 | Facilitation |
| 1400 | NO DEM | Control | YES | Subordinate | 2 | 0 | 0 | NA | N | NA | 0 | No Interaction |
| 1421 | NO DEM | Control | NO | Dominant | 2 | 0 | 0 | NA | N | NA | 0 | No Interaction |
| 1162 | NO DEM | Control | NO | Dominant | 2 | 0 | 0 | 12 | N | NA | 10.25 | Competition |
| 1143 | NO DEM | Control | NO | Subordinate | 2 | 0 | 0 | 23 | N | NA | 1.67 | Competition |
| 1054 | 1411 | Observer | NO | Subordinate | 2 | 41.11 | 2 | NA | N | NA | 0 | No Interaction |
| 1031 | 1411 | Observer | YES | Dominant | 2 | 97.22 | 2 | NA | N | NA | 0 | No Interaction |
| 1134 | 1411 | Observer | NO | Subordinate | 2 | 62.00 | 3 | 99 | N | NA | 2.54 | Cooperation |
| 1113 | 1411 | Observer | NO | Dominant | 2 | 62.00 | 3 | 4 | N | NA | 8.58 | Cooperation |
| 0920 | 1311 | Observer | NO | Subordinate | 2 | 19.44 | 2 | 0 | N | NA | 0 | No Interaction |
| 0951 | 1311 | Observer | NO | Dominant | 2 | 50.55 | 2 | NA | N | NA | 2.83 | No Interaction |
| 1408 | 1411 | Observer | YES | Dominant | 2 | 69.44 | 3 | 42 | N | NA | 0.33 | No Interaction |
| 1423 | 1411 | Observer | NO | Subordinate | 2 | 66.11 | 2 | 0 | N | NA | 1.25 | No Interaction |
| 1410 | 1311 | Observer | YES | Subordinate | 2 | 12.31 | 3 | NA | N | NA | 0 | No Interaction |
| 1403 | 1311 | Observer | YES | Dominant | 2 | 38.98 | 3 | 0 | Y | 378 | 29.63 | No Interaction |
| 1230 | 1311 | Observer | NO | Neutral | 2 | 47.83 | 2 | 5 | N | NA | 4.08 | Competition |
| 1241 | 1311 | Observer | NO | Neutral | 2 | 51.55 | 2 | 8 | N | NA | 2.17 | Competition |
| 1422 | 1411 | Observer | NO | Subordinate | 2 | 7.92 | 2 | 18 | N | NA | 6.67 | No Interaction |
| 1413 | 1411 | Observer | YES | Dominant | 2 | 64.70 | 3 | 0 | Y | 228 | 58.57 | No Interaction |
| 1220 | 1411 | Observer | NO | Neutral | 2 | 32.27 | 2 | 56 | N | NA | 0.67 | No Interaction |
| 1201 | 1411 | Observer | NO | Neutral | 2 | 66.18 | 3 | NA | N | NA | 0 | No Interaction |
| 1210 | 1311 | Observer | NO | Subordinate | 2 | 67.43 | 3 | NA | N | NA | 0 | No Interaction |
| 1251 | 1311 | Observer | NO | Dominant | 2 | 62.11 | 3 | 0 | N | NA | 2.18 | No Interaction |
| 1070 | 1311 | Observer | NO | Neutral | 2 | 37.65 | 3 | 32 | N | NA | 8.03 | Competition |
| 1033 | 1311 | Observer | YES | Neutral | 2 | 44.71 | 3 | 18 | N | NA | 7.58 | Competition |
| 0900 | NO DEM | Control | NO | Subordinate | 3 | 0 | 0 | 56 | N | NA | 1.18 | No Interaction |
| 08063 | NO DEM | Control | NO | Dominant | 3 | 0 | 0 | NA | N | NA | 0 | No Interaction |
| 0950 | NO DEM | Control | NO | Neutral | 3 | 0 | 0 | 0 | N | NA | 2.42 | No Interaction |
| 1141 | NO DEM | Control | NO | Neutral | 3 | 0 | 0 | NA | N | NA | 0 | No Interaction |
| 1400 | NO DEM | Control | YES | Subordinate | 3 | 0 | 0 | NA | N | NA | 0 | No Interaction |
| 1421 | NO DEM | Control | NO | Dominant | 3 | 0 | 0 | NA | N | NA | 0 | No Interaction |
| 1162 | NO DEM | Control | NO | Dominant | 3 | 0 | 0 | 28 | N | NA | 2.67 | Competition |
| 1143 | NO DEM | Control | NO | Subordinate | 3 | 0 | 0 | 39 | N | NA | 9.45 | Competition |
| 1054 | 1411 | Observer | NO | Subordinate | 3 | 37.12 | 1 | NA | N | NA | 0 | No Interaction |
| 1031 | 1411 | Observer | YES | Dominant | 3 | 98.56 | 3 | 25 | N | NA | 1.98 | No Interaction |
| 1134 | 1411 | Observer | NO | Subordinate | 3 | 32.63 | 2 | 198 | N | NA | 2.33 | Facilitation |
| 1113 | 1411 | Observer | NO | Dominant | 3 | 56.87 | 3 | 6 | N | NA | 12.17 | Facilitation |
| 0920 | 1311 | Observer | NO | Subordinate | 3 | 17.44 | 1 | 212 | N | NA | 5.56 | Facilitation |
| 0951 | 1311 | Observer | NO | Dominant | 3 | 43.78 | 3 | 0 | N | NA | 7.89 | Facilitation |
| 1408 | 1411 | Observer | YES | Dominant | 3 | 85.42 | 3 | 0 | N | NA | 2.17 | No Interaction |
| 1423 | 1411 | Observer | NO | Subordinate | 3 | 39.53 | 2 | 47 | N | NA | 3.92 | No Interaction |
| 1410 | 1311 | Observer | YES | Subordinate | 3 | 16.87 | 1 | NA | N | NA | 0 | No Interaction |
| 1403 | 1311 | Observer | YES | Dominant | 3 | 100 | 3 | 0 | Y | 166 | 33.33 | No Interaction |
| 1230 | 1311 | Observer | NO | Neutral | 3 | 33.72 | 2 | 3 | N | NA | 4.75 | Competition |
| 1241 | 1311 | Observer | NO | Neutral | 3 | 22.35 | 1 | 6 | N | NA | 1.67 | Competition |
| 1422 | 1411 | Observer | NO | Subordinate | 3 | 47.50 | 3 | 12 | N | NA | 6.11 | No Interaction |
| 1413 | 1411 | Observer | YES | Dominant | 3 | 98.12 | 3 | 0 | Y | 169 | 61.67 | No Interaction |
| 1220 | 1411 | Observer | NO | Neutral | 3 | 25.01 | 2 | 56 | N | NA | 0.87 | Facilitation |
| 1201 | 1411 | Observer | NO | Neutral | 3 | 45.78 | 2 | 79 | N | NA | 1.12 | Facilitation |
| 1210 | 1311 | Observer | NO | Subordinate | 3 | 52.67 | 3 | 46 | N | NA | 0.15 | Cooperation |
| 1251 | 1311 | Observer | NO | Dominant | 3 | 48.22 | 2 | 56 | N | NA | 0.47 | Cooperation |
| 1070 | 1311 | Observer | NO | Neutral | 3 | 23.53 | 2 | 2 | N | NA | 4.50 | Competition |
| 1033 | 1311 | Observer | YES | Neutral | 3 | 60.98 | 3 | 0 | N | NA | 7.67 | Competition |
| 0900 | NO DEM | Control | NO | Subordinate | 4 | 0 | 0 | 723 | N | NA | 0.78 | No Interaction |
| 08063 | NO DEM | Control | NO | Dominant | 4 | 0 | 0 | NA | N | NA | 0 | No Interaction |
| 0950 | NO DEM | Control | NO | Neutral | 4 | 0 | 0 | NA | N | NA | 0 | No Interaction |
| 1141 | NO DEM | Control | NO | Neutral | 4 | 0 | 0 | NA | N | NA | 0 | No Interaction |
| 1400 | NO DEM | Control | YES | Subordinate | 4 | 0 | 0 | NA | N | NA | 0 | No Interaction |
| 1421 | NO DEM | Control | NO | Dominant | 4 | 0 | 0 | NA | N | NA | 0 | No Interaction |
| 1162 | NO DEM | Control | NO | Dominant | 4 | 0 | 0 | NA | N | NA | 0 | No Interaction |
| 1143 | NO DEM | Control | NO | Subordinate | 4 | 0 | 0 | NA | N | NA | 0 | No Interaction |
| 1054 | 1411 | Observer | NO | Subordinate | 4 | 12.26 | 0 | NA | N | NA | 0 | No Interaction |
| 1031 | 1411 | Observer | YES | Dominant | 4 | 58.68 | 2 | NA | N | NA | 0 | No Interaction |
| 1134 | 1411 | Observer | NO | Subordinate | 4 | 26.52 | 1 | 156 | N | NA | 0.33 | Cooperation |
| 1113 | 1411 | Observer | NO | Dominant | 4 | 35.47 | 2 | 134 | N | NA | 1.12 | Cooperation |
| 0920 | 1311 | Observer | NO | Subordinate | 4 | 38.56 | 2 | 349 | N | NA | 2.26 | Facilitation |
| 0951 | 1311 | Observer | NO | Dominant | 4 | 42.19 | 3 | 225 | N | NA | 3.12 | Facilitation |
| 1408 | 1411 | Observer | YES | Dominant | 4 | 52.67 | 3 | 7 | N | NA | 5.42 | No Interaction |
| 1423 | 1411 | Observer | NO | Subordinate | 4 | 6.67 | 1 | 15 | N | NA | 1.00 | No Interaction |
| 1410 | 1311 | Observer | YES | Subordinate | 4 | 0 | 0 | NA | N | NA | 0 | No Interaction |
| 1403 | 1311 | Observer | YES | Dominant | 4 | 38.55 | 3 | 0 | Y | 157 | 60.00 | No Interaction |
| 1230 | 1311 | Observer | NO | Neutral | 4 | 27.37 | 2 | 0 | N | NA | 1.75 | Facilitation |
| 1241 | 1311 | Observer | NO | Neutral | 4 | 32.63 | 3 | 472 | N | NA | 0.67 | Facilitation |
| 1422 | 1411 | Observer | NO | Subordinate | 4 | 61.11 | 2 | NA | N | NA | 0 | No Interaction |
| 1413 | 1411 | Observer | YES | Dominant | 4 | 97.23 | 3 | 0 | Y | 192 | 41.67 | No Interaction |
| 1220 | 1411 | Observer | NO | Neutral | 4 | 12.18 | 1 | 0 | N | NA | 10.12 | Competition |
| 1201 | 1411 | Observer | NO | Neutral | 4 | 22.26 | 2 | 12 | N | NA | 3.33 | Competition |
| 1210 | 1311 | Observer | NO | Subordinate | 4 | 23.54 | 2 | 98 | N | NA | 2.26 | Cooperation |
| 1251 | 1311 | Observer | NO | Dominant | 4 | 33.98 | 2 | 125 | N | NA | 4.49 | Cooperation |
| 1070 | 1311 | Observer | NO | Neutral | 4 | 15.91 | 1 | 0 | N | NA | 5.67 | Competition |
| 1033 | 1311 | Observer | YES | Neutral | 4 | 35.27 | 2 | 8 | N | NA | 4.17 | Competition |
| 0900 | NO DEM | Control | NO | Subordinate | 5 | 0 | 0 | NA | N | NA | 0 | No Interaction |
| 08063 | NO DEM | Control | NO | Dominant | 5 | 0 | 0 | NA | N | NA | 0 | No Interaction |
| 0950 | NO DEM | Control | NO | Neutral | 5 | 0 | 0 | NA | N | NA | 0 | No Interaction |
| 1141 | NO DEM | Control | NO | Neutral | 5 | 0 | 0 | NA | N | NA | 0 | No Interaction |
| 1400 | NO DEM | Control | YES | Subordinate | 5 | 0 | 0 | NA | N | NA | 0 | No Interaction |
| 1421 | NO DEM | Control | NO | Dominant | 5 | 0 | 0 | NA | N | NA | 0 | No Interaction |
| 1162 | NO DEM | Control | NO | Dominant | 5 | 0 | 0 | NA | N | NA | 0 | No Interaction |
| 1143 | NO DEM | Control | NO | Subordinate | 5 | 0 | 0 | NA | N | NA | 0 | No Interaction |
| 1054 | 1411 | Observer | NO | Subordinate | 5 | 8.23 | 0 | NA | N | NA | 0 | No Interaction |
| 1031 | 1411 | Observer | YES | Dominant | 5 | 78.54 | 3 | 46 | N | NA | 2.12 | No Interaction |
| 1134 | 1411 | Observer | NO | Subordinate | 5 | 21.85 | 1 | NA | N | NA | 0 | No Interaction |
| 1113 | 1411 | Observer | NO | Dominant | 5 | 39.26 | 1 | NA | N | NA | 0 | No Interaction |
| 0920 | 1311 | Observer | NO | Subordinate | 5 | 40.83 | 3 | NA | N | NA | 0 | No Interaction |
| 0951 | 1311 | Observer | NO | Dominant | 5 | 33.33 | 3 | NA | N | NA | 0 | No Interaction |
| 1408 | 1411 | Observer | YES | Dominant | 5 | 100.00 | 3 | 0 | N | NA | 3.92 | No Interaction |
| 1423 | 1411 | Observer | NO | Subordinate | 5 | 3.33 | 0 | NA | N | NA | 0 | No Interaction |
| 1410 | 1311 | Observer | YES | Subordinate | 5 | 12.17 | 0 | NA | N | NA | 0 | No Interaction |
| 1403 | 1311 | Observer | YES | Dominant | 5 | 33.33 | 1 | 0 | Y | 138 | 58 | No Interaction |
| 1230 | 1311 | Observer | NO | Neutral | 5 | 27.22 | 2 | 11 | N | NA | 4.92 | Competition |
| 1241 | 1311 | Observer | NO | Neutral | 5 | 17.78 | 1 | 14 | N | NA | 3.17 | Competition |
| 1422 | 1411 | Observer | NO | Subordinate | 5 | 24.17 | 1 | NA | N | NA | 0 | No Interaction |
| 1413 | 1411 | Observer | YES | Dominant | 5 | 75.00 | 3 | 0 | Y | 146 | 41.90 | No Interaction |
| 1220 | 1411 | Observer | NO | Neutral | 5 | 3.15 | 1 | 0 | N | NA | 0.67 | Competition |
| 1201 | 1411 | Observer | NO | Neutral | 5 | 15.16 | 2 | 2 | N | NA | 1.67 | Competition |
| 1210 | 1311 | Observer | NO | Subordinate | 5 | 37.46 | 3 | NA | N | NA | 0.00 | No Interaction |
| 1251 | 1311 | Observer | NO | Dominant | 5 | 45.66 | 3 | NA | N | NA | 0.00 | No Interaction |
| 1070 | 1311 | Observer | NO | Neutral | 5 | 24.33 | 1 | NA | N | NA | 0.00 | No Interaction |
| 1033 | 1311 | Observer | YES | Neutral | 5 | 38.17 | 2 | 0 | N | NA | 4.25 | No Interaction |
