# Supplementary material for: Persistence and conspecific observations improve problem-solving abilities of coyotes
Source: PLoS One. 2019 Jul 10;14(7):e0218778. doi: 10.1371/journal.pone.0218778 (PMC6619663; doi:10.1371/journal.pone.0218778)
Supplement: S7 Table — (DOCX) [file pone.0218778.s007.docx]

| **S7 Table.** Raw data from post-trial eating task during study 3. | | | | | |
| --- | --- | --- | --- | --- | --- |
| **Coyote ID** | **Demonstrator ID** | **Treatment Group** | **Social Rank** | **Time to eat** | **First to eat** |
| 1054 | 1411 | Observer | Subordinate | 6.18 | N |
| 1031 | 1411 | Observer | Dominant | 6.02 | Y |
| 1134 | 1411 | Observer | Subordinate | 5.32 | N |
| 1113 | 1411 | Observer | Dominant | 5.06 | Y |
| 1408 | 1411 | Observer | Dominant | 1.36 | Y |
| 1423 | 1411 | Observer | Subordinate | 2.01 | N |
| 1422 | 1411 | Observer | Subordinate | 4.36 | N |
| 1413 | 1411 | Observer | Dominant | 0.33 | Y |
| 1220 | 1411 | Observer | Neutral | 23.18 | N |
| 1201 | 1411 | Observer | Neutral | 23.17 | Y |
| 0920 | 1311 | Observer | Subordinate | 5.47 | N |
| 0951 | 1311 | Observer | Dominant | 5.08 | Y |
| 1410 | 1311 | Observer | Subordinate | 2.46 | N |
| 1403 | 1311 | Observer | Dominant | 0.22 | Y |
| 1210 | 1311 | Observer | Subordinate | 17.56 | Y |
| 1251 | 1311 | Observer | Dominant | 19.32 | N |
| 1230 | 1311 | Observer | Neutral | 9.19 | Y |
| 1241 | 1311 | Observer | Neutral | 10.15 | N |
| 1070 | 1311 | Observer | Neutral | 18.46 | Y |
| 1033 | 1311 | Observer | Neutral | 19.11 | N |
| 0900 | NO DEM | Control | Subordinate | 9.12 | N |
| 08063 | NO DEM | Control | Dominant | 8.34 | Y |
| 0950 | NO DEM | Control | Neutral | 7.02 | N |
| 1141 | NO DEM | Control | Neutral | 6.55 | Y |
| 1400 | NO DEM | Control | Subordinate | 17.32 | N |
| 1421 | NO DEM | Control | Dominant | 16.49 | Y |
| 1162 | NO DEM | Control | Subordinate | 24.54 | Y |
| 1143 | NO DEM | Control | Dominant | 26.18 | N |
