# Supplementary material for: Persistence and conspecific observations improve problem-solving abilities of coyotes
Source: PLoS One. 2019 Jul 10;14(7):e0218778. doi: 10.1371/journal.pone.0218778 (PMC6619663; doi:10.1371/journal.pone.0218778)
Supplement: S1 Fig — All individuals used in both studies 2 and 3 were considered. Using a Chi-square test of independence, we found that sex and social rank were highly dependent, for individuals used in both studies (P = 0.0002 and P = 0.027, respectively for study 2 and study 3), males being more dominant than females. (DOCX) [file pone.0218778.s008.docx]

**S1 Fig**. Distribution of the three classes of social rank between sexes. All individuals used in both studies 2 and 3 were considered. Using a Chi-square test of independence, we found that sex and social rank were highly dependent, for individuals used in both studies (*P* = 0.0002 and *P* = 0.027, respectively for study 2 and study 3), males being more dominant than females.

*
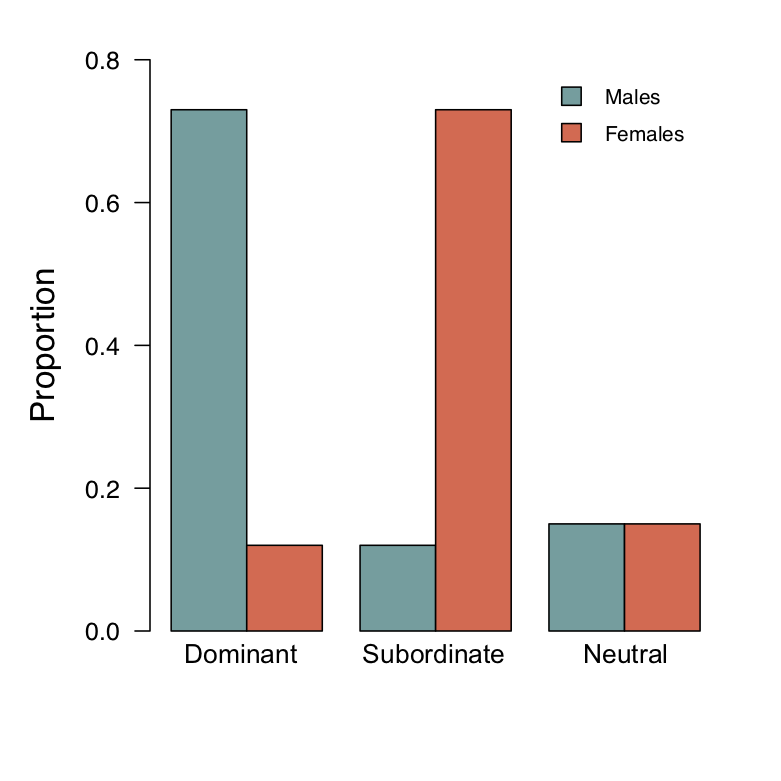
*
